# The revitalization of endangered heritage buildings in developing countries: A decision-making framework for investment and determining the highest and best use in Egypt

**DOI:** 10.12688/f1000research.135214.1

**Published:** 2023-07-24

**Authors:** Mohanned Selim, Adham Abulnour, Sally Eldeeb

**Affiliations:** 1 Architectural Engineering & Environmental Design, Arab Academy for Science Technology and Maritime Transport, Alexandria, Alexandria Governorate, 21913, Egypt

**Keywords:** Adaptive Reuse, Heritage Buildings, Mixed-Use, Real-estate Development, Decision-making parameters, Stakeholders, Investment, Highest and best use

## Abstract

**Background:** Egypt’s major cities have been losing heritage and historical buildings due to neglect and misuse, prompting non-governmental organizations, academic institutions, and researchers to advocate for adaptive reuse strategies to preserve the cities’ heritage and identity. Adaptive reuse involves changing, modifying, or reusing a space based on community needs, business model, location, and proximity to facilities and services. Heritage buildings offer many tangible and intangible benefits that enhance financial returns, making them challenging but feasible and attractive for investors who value authenticity, uniqueness, and sustainability.

**Methods:** This study examines how market value, acquisition opportunities, target clients, age groups, and socioeconomic status affect decision-making. A comparative analysis of three buildings in the Egyptian cities of Alexandria and Cairo is utilized to establish development guidelines and decision-making parameters that significantly impact project design and building functions to determine the highest and best use. In order to complete this study, AutoCAD by Autodesk was used for 2D drawings, SketchUp by Trimble for 3D models, Adobe Photoshop for diagram presentation, and Microsoft Office for tables and diagrams.

**Results:** The comparative analysis provided valuable insights into the adaptive reuse of heritage buildings in developing countries. Findings highlighted how cultural heritage preservation could foster socioeconomic development. Key success factors included stakeholder and community engagement, financial viability, and architectural compatibility. The decision-making framework provides a practical tool for evaluating heritage building reuse.

**Conclusions:** The analysis illustrates successful reuse strategies and considerations. Decision-making frameworks and tools offer practical guidance for future investments and decisions. These findings affect heritage conservation and urban development policymakers, planners, and investors. Stakeholders can make informed decisions and implement strategies to preserve cultural and environmental value by realizing challenges and opportunities. This study hopes to inspire more research and help preserve and revitalize heritage buildings in developing countries, preserving their cultural and socioeconomic value.

**List of abbreviations**
AR: Adaptive Reuse
AVI: Avierino Building
B&B: Bed and Breakfast
BOQ: Bill of Quantities
BUA: Built-Up Area
CBD: Commercial Business District
F&B: Food and Beverage
HBU: Highest and best use
HVAC: Heating, Ventilation and Air-Conditioning
LV: Little Venice Building
MEP: Mechanical, Electrical, and Plumbing
MUD: Mixed-Use Development
NGO: Non-Governmental Organization
OUZ: Ouzonnian Building
RCP: Reflected Ceiling Plan
ROA: Return on Asset
ROI: Return on Investment
VRF: Variable Refrigerant Flow

## Introduction

Adaptation is a technique for prolonging the usable life of structures through a mix of modification and conversion ( Kohler and Hassler, 2002). Adaptive reuse of historic structures or modifying the original use of a heritage building to meet new conditions or demands and so reusing it, is critical to the sustainable growth of communities by avoiding demolition and rebuilding procedures ( Aplin, 2002). Reusing properties may provide important community resources, significantly cut land purchase and building costs, rejuvenate existing communities, and restrict sprawl ( Godwin, 2011). Preserving the integrity of an existing building, in particular, may reduce material, transportation, and energy use, as well as pollution, and so contribute significantly to reducing carbon dioxide emissions and improving sustainability ( Bullen, 2007; Chung, 2009).

This realization has become part of redevelopment strategies and visions in countries all over the world ( Mısırlısoya and Günc, 2016), and this can be witnessed in Egypt nowadays after several non-governmental organizations (NGOs) have highlighted the issue of heritage building demolition ( Power, 2008); the government, alongside a limited number of private sector companies, started to implement adaptive reuse strategies in downtown Cairo and Alexandria as the existing governmental facilities and institutions are all being relocated to new settlements and cities such as the New Administrative Capital.

### Research aim and objectives

This research was created with the goal of providing a framework for developing heritage assets and criteria for investment in order to help investors, designers, and stakeholders prepare for projects and measure their performance ( Bond, 2011; Steinberg, 1996). Identifying the factors that must be taken into consideration when deciding to invest in heritage properties or related projects and defining the tangible and non-tangible parameters to facilitate the decision-making process for the highest and best use of each space in a property is essential for developers in order to simplify the process and make it clear for all stakeholders that investing in heritage is a sustainable investment that is feasible and profitable ( Shankland, 1975; Yung and Chan, 2012).

## Methods

This study performs a thorough analysis of different contemporary literature focusing on adaptive reuse, valorization methods of built heritage, and mixed-use development. This literature review forms the basis for a proposed conceptual framework and technical approach that are formed from the researcher’s experience in the field of adaptive reuse as practiced in the cities of Alexandria and Cairo.

In the development of the decision-making framework and investment criteria, several tools and software were instrumental in the process. AutoCAD by Autodesk ( https://www.autodesk.co.uk/) played a crucial role in creating 2D drawings, providing precise representations of the building’s existing conditions and proposed modifications (students and educators can access a free version, while commercial use requires a subscription). SketchUp by Trimble ( https://www.sketchup.com/) was used for generating 3D models, enabling a more immersive and realistic visualization of the adaptive reuse designs (free and pro versions are available). Photoshop by Adobe ( https://www.adobe.com/uk/) was utilized for creating visually appealing diagrams and presentations that effectively conveyed project concepts (subscription-based, including a free trial). Additionally, Microsoft Office applications such as PowerPoint and Excel were utilized for organizing data, generating tables, and creating informative diagrams (free versions are available for students and educators, while commercial use requires a subscription). These software tools collectively enabled the author to formulate a comprehensive framework, supporting the successful execution of adaptive reuse projects for heritage buildings. Free alternatives for AutoCAD include FreeCAD ( https://www.freecad.org/) and LibreCAD ( https://librecad.org/), while alternatives for Photoshop include GIMP ( https://www.gimp.org/) and Krita ( https://krita.org/en/). For 3D modeling, Blender ( https://www.blender.org/) serves as a free alternative to SketchUp. LibreOffice ( https://www.libreoffice.org/) and Google Docs are free alternatives to Microsoft Office applications like PowerPoint and Excel.

The first part of this section discusses the development process of the adaptive reuse of heritage buildings based on existing projects and completed adaptive reuse (AR) scenarios in Egypt. The development process encompasses several vital steps that contribute to its overall detailed execution. Firstly, asset evaluation is conducted to thoroughly assess the physical condition, historical significance, and potential of the heritage building for reuse. This step provides a comprehensive understanding of the building’s unique features and challenges, aiding in decision-making for the adaptive reuse project. The next step involves the identification of programs and functions, where the specific uses and activities to be incorporated into the building are determined. This step ensures that the AR aligns with the desired goals and objectives of the project. Subsequently, the development process moves towards the formulation of a business model and stakeholder agreements, addressing the financial aspects, partnerships, and responsibilities associated with the project. Brand and identity creation are then emphasized to establish a distinct image and positioning for the AR development. In the design phase, architectural and engineering plans are formulated, incorporating both the historical elements and modern requirements of the project. Tendering and contractor selection follow, involving the procurement of necessary services and materials. Finally, the operation and evaluation phase ensures the effective management and ongoing assessment of the adaptive reuse project to maintain its success and address any necessary improvements.

The development of the decision-making parameters process is then supported by identifying stakeholders and key players in the industry and analyzing the nature of those entities as well as their different roles throughout the process ( Wang and Zeng, 2010). The previous is supported by multiple case studies where adaptive reuse was implemented and achieved completely or partially in order to finally come up with simple guidelines and a framework that helps developers and other stakeholders in the decision-making process for the components and activities incorporated in a project. Those projects were utilized by the researcher as a reference due to multiple factors such as functional and economic viability, preservation of heritage value, community engagement and stakeholder collaboration, and finally, the recognition of the local community ( Merlino, 2018).

The comparative analysis followed multiple stages in order to develop a thorough evaluation tool which then ultimately led to the decision-making framework that is meant to help stakeholders decide on the highest and best use. The preliminary evaluation of each building was conducted by considering several decision-making parameters. The first parameter, location, was measured based on its proximity to key amenities, transportation networks, and potential target markets. Accessibility, the second parameter, was graded by assessing the ease of reaching the property through various modes of transportation. The surrounding context was evaluated by analyzing neighboring spaces and buildings with similar or complementary functions, determining whether they could contribute to the success of the mixed-use development. The architectural description and features were examined to determine the property’s aesthetic appeal, historical significance, and potential for adaptive reuse. Spatial challenges and restrictions, such as heights and areas, were measured to assess the feasibility of incorporating desired functions within the existing structure. Existing services and facilities were evaluated to determine the extent of infrastructure that could be utilized or upgraded. Available spaces within the property were assessed to understand the potential for accommodating different uses. Lastly, socioeconomic analysis played a crucial role in grading the property based on the demographics and economic conditions of the surrounding area. This comprehensive evaluation of each parameter provided a brief preliminary assessment of the heritage buildings through a brief description and a grading which ranges between High, Average, and Low based on the aforementioned measurement factors.

The researcher then developed an evaluation system from the data and analyses gathered throughout this study as shown in Table 1. This method follows a simple evaluative approach for each of the aforementioned parameters based on multiple factors (sub-parameters) that have an impact on the main parameter. The researcher then provides a total grade for each main parameter, which will then be used in the following step of the evaluation system and will also be used to assist with the comparison between the three analyzed case studies, ultimately formulating the general guidelines and decision-making criteria regarding the function, type, category, and scale. The grading was intentionally simplified in order to make the process easy for application to other buildings in any AR project by different stakeholders, but mainly by investors and producers. Each sub-parameter is given a value from 0 to 2. The 0 represents a low evaluation of the parameter, the 1 represents an average evaluation, and the 2 represents a high evaluation.

**Table 1.** Table demonstrates the detailed evaluation of heritage buildings based on the developed parameters and sub-parameters proposed by the author. Source: Author, 2023.

Main parameter	Evaluation based on sub-parameters grading: Low (0) – Average (1) – High (2)
Location	Views
	Historical Significance (Location Attributes) (Touristic or commercial)
	Minimum Environmental Impact (Noise or other pollution level)
	Market Demand
Accessibility	Diverse Modes of Transport (Private cars and vehicles)
	Connected to Main Roads
	Public Transport (Train, Tram, Metro, Buses, Taxis, etc.)
	Proximity of Parking and Facilities
Surrounding context	Proximity to Amenities
	Safety
	Market Value
	Engagement with Surrounding Community (Vitality)
Architectural description	Building Condition
	Historical Significance (Architect & Building)
	Overall Features & Ornaments
	Façades State & Features (Minimum Transgressions)
	Old Elements of Interior Spaces still intact (Floors, Windows, etc.)
	Category of Building (Heritage listing, building is protected and flexible for development)
Spatial characteristics	Structure System (Type, state, and flexibility of opening spaces)
	Open Spaces Availability
	Heights
	Sizes of Openings (Windows of Facades and Courts)
Facilities	State and Scale of Available Infrastructure (MEP)
	Elevators and Service Cores
	State and Scale of Courts
	Roof and Basement availability and state
Available spaces	Legal Situation (Percentage of Available spaces and types, old or new law rentals)
	Ground Floor Area & Availability for Retail/Commercial uses
	Roof Availability and Readiness for Development
	Basement Availability and Readiness for Development
	Connectivity of Spaces
Socioeconomic analysis	Economic Conditions of local community
	Cultural Level
	Social Level
	Job Growth (Increasing Level of Job Opportunities)
	Population levels

The researcher performs this assessment by analyzing each parameter and how it affects the four mentioned aspects (function, scale, type, and category) and follows this step by adding the values of all the parameters that have an impact on each of the four aspects, as shown in Table 2 below, which demonstrates which aspect each and every parameter has an impact on. This total value is then converted to a percentage in order to make its evaluation more efficient and legible in the decision regarding each aspect or step.

**Table 2.** Table demonstrates the impact of each parameter on the four aspects or decision-making phases when developing AR projects. Source: Author, 2023.

Parameter	Direct impact on: (Function – Scale – Type – Category)	Description - Function: Retail/Commercial, Hospitality, Offices, Arts & Culture - Scale: Large, Medium, Small - Type: Type of each Function (i.e., Hotel or Serviced Apartments) - Category: High, Average, Low (Level of finishing, furniture, and appliances based on target users)
Location	Function – Type – Category	Has a direct impact on the decision of which function or new use is most suitable to be incorporated in AR developments since a building’s location is a critical parameter that guides this decision. An example of this is when a building is in a prime waterfront location, it has a higher potential to incorporate hospitality as a main function; if the building is in a CBD, it has a potential for more office spaces, etc. This also impacts the decision of the type of function and category of this type as the following parameter.
Accessibility	Type – Category	Has a direct impact on the type of project decision regarding each decided function since the aspects related to accessibility can allow incorporating certain functions and activities while hindering others, such as the availability of diverse methods of public transportation such as buses and metro stations. This grants more access to certain types of traffic, which could require certain services and facilities such as banks, F&B, and other retail services. Also, the availability of parking spaces identifies the function and type, as certain uses require parking facilities, such as luxurious hotels, while boutique hotels might not need it as much.
Surrounding context	Function – Category	Has a direct impact on function since the surrounding amenities help guide developers in understanding market demand and the target users of the project, leading to a clear decision of what the surrounding area and community require or need. Also, the surrounding context helps form a clear idea of the category of the project as passersby and potential users are identified. For example, if this building is located in a touristic area where backpackers and younger generations of users visit, a short-term stay hospitality service could be provided with a medium-level category of finishes and furniture. Also, such an area could require the availability of simple, yet functional coworking spaces as well as F&B options.
Architectural description	Type – Category	Has a direct impact on the decision regarding the most suitable type of each function. If the new function is hospitality, this parameter guides the developers in deciding whether it should be a luxurious hotel chain, a boutique hotel, a hostel, or an Airbnb stay. Also, this consequently impacts the category of finishes and furniture based on knowing the targeted users and clients.
Spatial characteristics	Function – Scale	Has a direct impact on function, such as in the case of smaller spaces, this could limit the options for new activities. If a project requires a large space with an open plan, such as a coworking space or exhibition area, and such a space is unavailable due to structural limitations, the function will inevitably change. Heights also impact the scale of the project as they provide more spaces that could be utilized for more functions or extensions.
Facilities	Function – Scale	Has a direct impact on the function Since the facilities in the building are critical for any introduced activity, in cases where the building does not have sufficient facilities such as courts and MEP infrastructure, it might be harder for the developer to incorporate fully dedicated F&B services. Also, in cases where the building doesn’t have enough service cores with elevator access for users, especially the disabled visitors, functions with high traffic might not be suitable for said space or building. Consequently, this parameter defines and imposes the scale of the project, as the facilities in a building are correlated to the scale of the introduced uses.
Available Spaces	Function – Scale – Type	Has a direct impact on the introduced function; for example, the availability of lower floors provides the opportunity for adding retail or commercial activities; this also impacts the scale of the project as in some cases the spaces available could be limited to a few apartments or floors, while in other cases the building could be fully available, allowing for larger scale projects. Finally, the type of project is impacted, such as in the aforementioned case of hospitality, where limited spaces would be an obstacle to incorporating a full-scale hotel project in the building, but a boutique hotel would be more suitable.
Socioeconomic Analysis	Function – Category	Has a direct impact on the function and category of the adaptive reuse project since the type of function should be suitable to the needs and status of the local community and potential users; a location with a simple, lower economic status would not be suitable for premium retail services where the traffic might not relate to or find use in such a service. Also, an area with a lower social level might require educational facilities and cultural centers to assist with revitalizing the community and raising its overall status.

After defining all the parameters that have an impact on each aspect as shown in Table 2 above, the researcher adds those values and concludes with the final guidelines that would potentially assist stakeholders involved in AR projects in heritage buildings with the decision-making process from start to finish regarding the highest and best use and optimum component mix to be incorporated in the building, as shown in Table 3 below, and applies those steps to the case studies. The aforementioned four steps are demonstrated as follows:

**Table 3.** Table demonstrates the four decision-making phases and the decision based on the overall evaluation percentage. Source: Author, 2023.

Step 1: Function	
**Percentage** (Sum value of all parameters with impact on Function)	**Function/Activity**
If 10 to 40%	Less potential for Mixed Use developments (MUD), more retail/commercial, including banks and telecom.
If 40 to 70%	Higher potential for MUD, more hospitality and Educational/Cultural.
If 70 to 100%	Maximum potential for MUD, more offices and coworking spaces, more F&B, and more Arts spaces and exhibitions.
Step 2: Scale	
**Percentage** (Sum value of all parameters with impact on Scale)	**Scale**
If 10 to 40%	Small
If 40 to 70%	Medium
If 70 to 100%	Large
Step 3: Type	
**Percentage** (Sum value of all parameters with impact on Type)	**Type of Function/Activity** Depending on each function, as shown in Table 4
If 10 to 40%	Targeting local and lower income tenants/users
If 40 to 70%	Targeting medium income users and younger generations of tourists and visitors
If 70 to 100%	Targeting higher income users and older generations of tourists and visitors
Step 4: Category	
**Percentage** (Sum value of all parameters with impact on Category)	**Category** (Level of finishes, furniture, and equipment)
If 10 to 40%	Low
If 40 to 70%	Average
If 70 to 100%	High

The recommended types previously mentioned as part of the four-step evaluation process are described in detail by the researcher with regards to every function, as shown below in Table 4. The table determines the general framework where each function will be decided considering the target users and based on the evaluation, where the percentages from 10 to 40 will mainly, but not exclusively, target locals of lower income, and percentages from 40 to 70 will mainly, but not exclusively, target medium-income users and younger generations of tourists and locals, and finally, percentages from 70 to 100 will mainly, but not exclusively, target higher-income users and older generations from the same group.

**Table 4.** Table demonstrates the recommended type of function based on the evaluation percentage. Source: Author, 2023.

Function	Percentage	Recommended type
Commercial/Retail/F&B	10 to 40%	Local vendors and workshops
	40 to 70%	Cafes and restaurants (mid-range and fast food), showrooms, banks and telecom
	70 to 100%	Fine dining, high-end brands, and magnet stores
Hospitality/Residential	10 to 40%	Residential low budget rentals and hostels
	40 to 70%	Boutique hotels, serviced apartments and B&B
	70 to 100%	Hotels, and high-end hospitality (unique concept hotels)
Offices and Coworking	10 to 40%	Individual offices for local tenants with different uses
	40 to 70%	Mid-range offices and coworking spaces (hot desks)
	70 to 100%	Representative (Rep) offices and high-end coworking spaces with communal areas and hubs
Art and Culture/Education	10 to 40%	Educational centers and institutions
	40 to 70%	Cultural centers, arts/music studios and spaces for young and rising artists
	70 to 100%	Premium art spaces and exhibitions for both rising and established artists

This methodology is proposed by the author in order to reach an ultimate decision regarding investment opportunities and determine the highest and best use of heritage buildings that are intended for adaptive reuse projects in Egypt and developing countries. The proposed system is generalized in order to make it simple to replicate, readapt, or possibly undergo further development, turning it into a digital tool that would facilitate the decision-making process for different stakeholders involved in the adaptive reuse industry.

### Development process

*Asset evaluation*

The first stage in the adaptive reuse process is the acquisition stage, when investors and developers start to search for unique properties and assets that suit their scope and development vision. These properties must have a special location that is easily accessible via diverse transportation methods ( Brandt, 2006). Also related to the location are the views of the building, the main landmarks, the surrounding activities, and the socioeconomic status of the community ( Browne, 2006).

After analyzing the location, the investors start to analyze the building from the inside, considering the available spaces, interior details, areas and heights, and existing services and facilities ( Rabun and Kelso, 2009). Furthermore, historical studies and documentation are made to identify the architect, architectural style, ornaments, and features in order to identify the original designs and restore them where applicable, which then creates a unique product and adds value to the asset ( NOUH, n.d.). Finally, structural reports are prepared by experts to ensure the building maintains its structural integrity and is worth the investment.

*Programs and functions identification*

After acquiring the building, the development process starts with its initial step of identifying the spaces within the property that will be developed within the company’s development pipeline.

**Figure 1.**
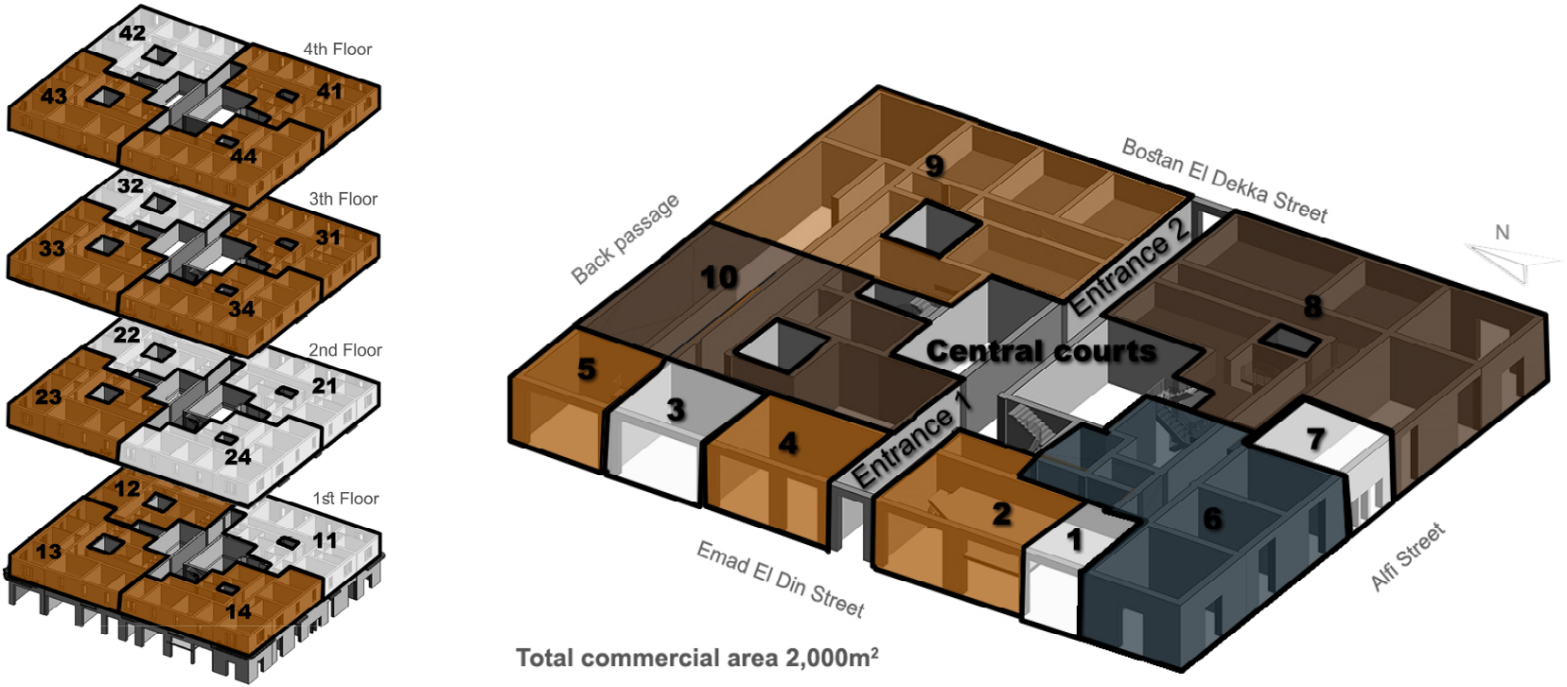
Diagram showing development vision and program of Emad El Din building, Cairo. **Source**: This figure has been reproduced with permission from Sigma Properties, 2019. Key: ● Unavailable Spaces ● leased old law (Unavailable) ● available (acquired) ● leased new law (available).

There are various cases regarding available spaces in adaptive reuse scenarios: the building could be a standalone building that is completely available and ready to be developed as a whole; the building could have whole floors that are empty and other floors that are rented or owned, as shown in Figure 1; or it could have separate spaces, such as apartments or stores, that are currently available or leased under a contract that is nearing an end. After that, it is required to perform market research to study the feasibility of a project and forecast the expected return on investment (ROI) and return on asset (ROA) to ensure the project’s success. Finally, based on all the aforementioned steps, the suitable component mix is decided, as well as the percentages of all the functions to be added to the asset ( Ribera *et al.,* 2020).

*Business model and stakeholder agreements*

In this phase, the developers, along with real estate advisors, property management companies, and business developers, start to search for suitable operators to manage and run the projects ( Menassa and Baer, 2014). Also, the project usually has an estimated budget that is defined based on the target users, categories of provided services, and types of functions.

*Brand and identity creation*

**Figure 2.**
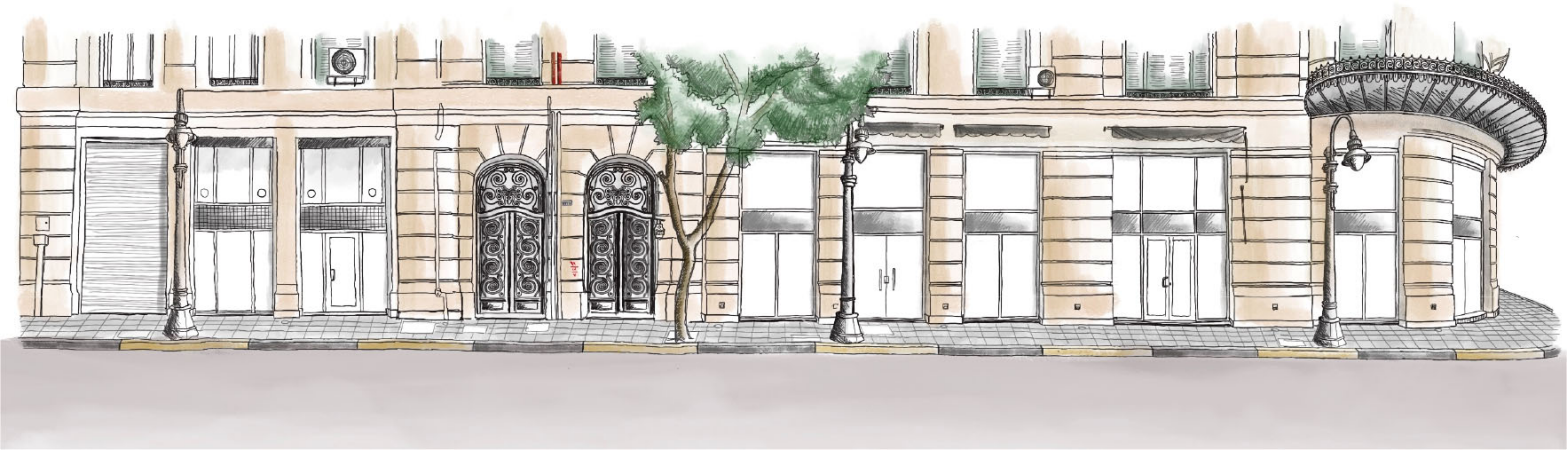
Sketch of Avierino building in Alexandria; for potential branding and marketing purposes. Source: Author, 2022.

In this phase, designers and marketing experts start creating a unique brand for the asset, as well as sub-brands for the available functions, to make it a destination where you can have a unique experience like no other. Part of this brand creation involves the procurement of artworks as shown in Figure 2, photos, and other visual materials that represent the building and portray its identity, which could then be used in websites, social media platforms, and investment and marketing presentations.

Also, the building’s story is highlighted; this story includes the architect, architectural features, and interior and exterior details, all of which play a major role in making the building stand out and deserve special recognition ( Abdeen and Ahmed, 2009). Furthermore, restoring lost features, removing transgressions, and preserving existing elements also adds value to the asset ( Megahed, 2009).

*Design phase*

**Figure 3.**
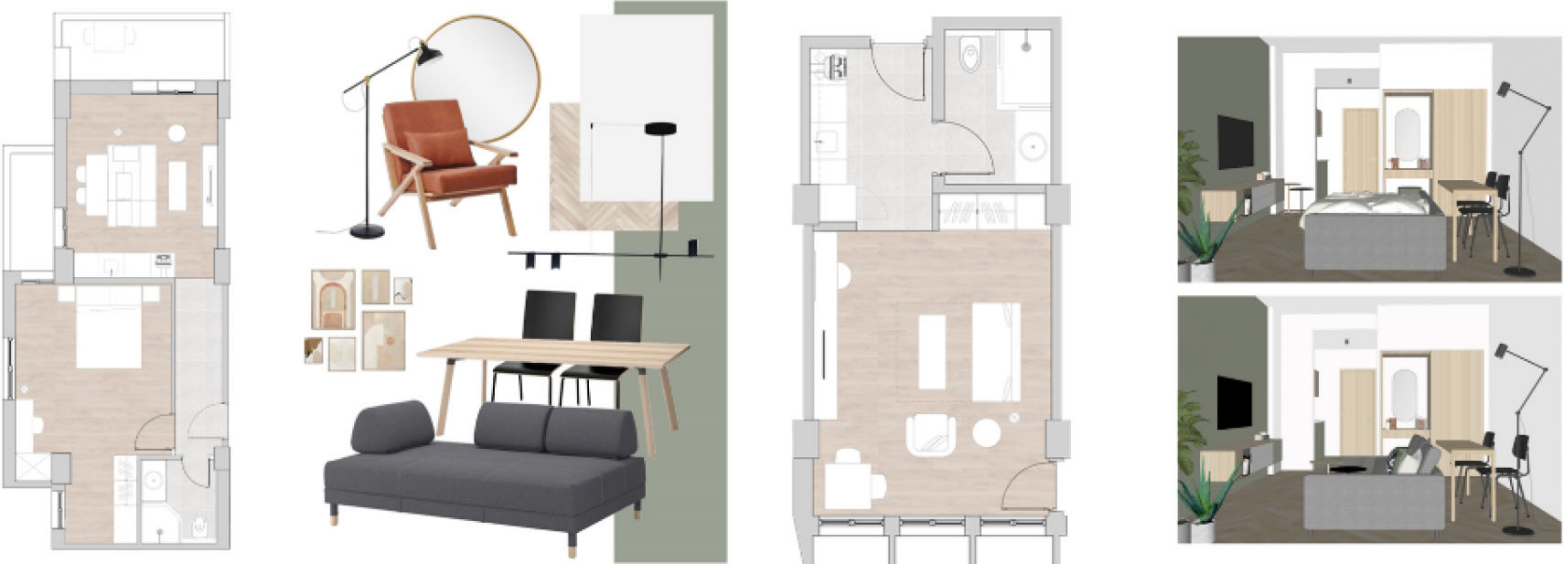
Plans of apartments and studios showing proposed furniture, materials, and finishes in Ouzonnian building, Cairo. Source: This figure has been reproduced with permission from Sigma Properties, 2021.

In this phase, the design studio starts working in coordination with the developer on the project’s design concept, showing the proposed style, design, inspiration, and overall look and feel of the space. Also, mood boards and material boards are then proposed, along with renders that help elaborate the proposed design, allowing the investor to have a clear image of what the space will look like after development as shown in Figure 3.

**Figure 4.**
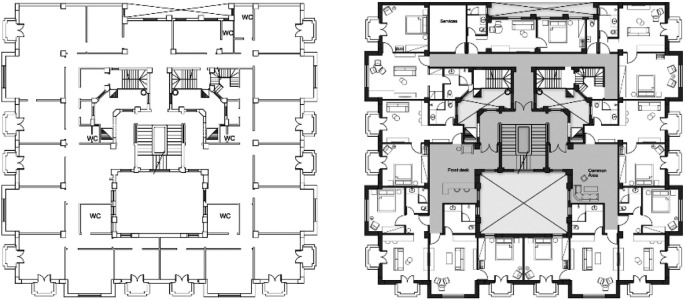
Drawings of floor plans before and after redesign and reuse. Source: This figure has been reproduced with permission from Sigma Properties, 2022.

After finalizing the designs, the design team starts preparing the drawings package. The drawings include furniture layouts, electromechanical and plumbing (MEP) plans, reflected ceiling plans (RCP), and demolition and addition drawings that illustrate the as-built plans as well as the proposed new design marking the required modifications as shown in Figure 4.

*Tendering and contractor selection*

In this stage, the design team and the project consultant, representing the investor, coordinate the preparation of the bill of quantities (BOQ) and vendor list based on the data prepared in the design phase to be presented to the contractor or various contractors for cost comparison and selection. During site work, the design team along with the consultant supervise the project and follow-up with the delivery of each phase of site work until the project is successfully delivered.

*Operation and evaluation*

In the final phase of the development process, following the successful delivery of the project, the operator initiates the pilot phase of the project. In this phase, the operator works on marketing and public outreach; this includes social media campaigns, public events, online advertisement, and other methods that help the project reach its target audience. This phase happens in coordination with the developer, operator, and design team in order to ensure a smooth pilot phase and work on any issues or modifications that might be required. After that, a major evaluation is made to measure the project’s performance in order to perform any optimizations to the project itself and to future projects ( Giles, 2005).

**Figure 5.**
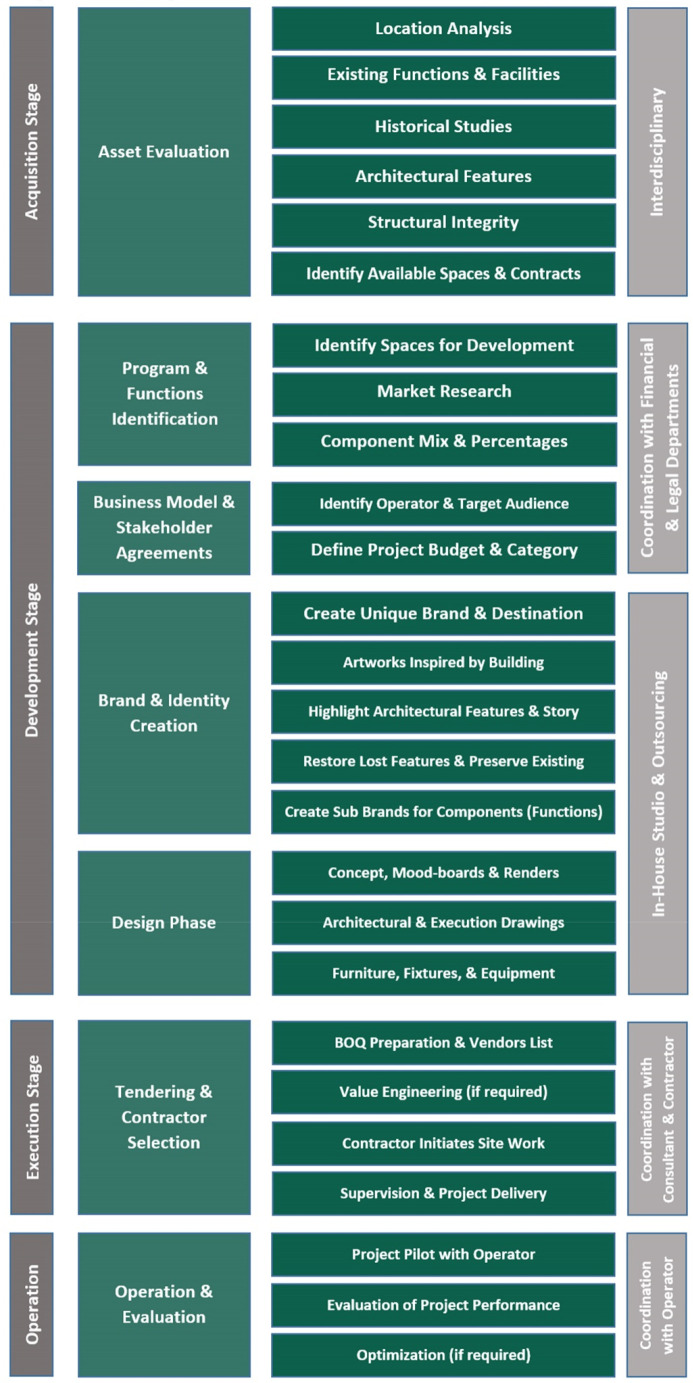
Summarized table showing Development process stages and involved stakeholders. Source: Author, 2022.

### Stakeholders identification

Most complicated adaptive reuse challenges require the collaboration of diverse stakeholders with divergent interests to realize mutually gratifying results. Eventually, the identification of important stakeholders and the collaborative rationale that exists between them in a decision-making process will be essential to the effective implementation of any strategy for sustainable development. In the majority of adaptive reuse decision-making scenarios, contradictory views, perspectives, interests, and resources exist among important parties ( Aigwi *et al.,* 2019). Therefore, it would be advantageous for all participants in an adaptive reuse decision-making process to understand who the other players are, how their interests are interconnected, and how the collaborative strategy operates ( Innes and Booher, 2010; *Department of Environment and Heritage*, 2004).

*Investors*

An adaptive reuse project could have investors from the government, the private sector, owners of historic buildings, financing groups, renters, foreign communities, and other developers.

*Producers*

The producer category includes all the players participating in the production of an adaptive reuse project, which includes the designers, structural engineers, heritage restoration experts, consultants, and contractors.

*Operators*

Includes local or international brands and chains representing any of the functions in a building; this includes hotels, offices, retail, food and beverage (F&B) chains, or independent entities.

*Regulators*

The regulator category includes all governmental institutions concerned with the governance of real estate development, laws, and permits. Also, entities that identify heritage assets, regulate their existing states, and approve their redevelopment.

*Users*

Members of the local community, visitors, original users (current tenants), or prospective tenants comprise this stakeholder category ( Bond, 2011).

### Comparative analysis

*Property assessment for mixed-use developments: decision-making parameters*

• Location
• Accessibility
• Surrounding context (neighboring spaces and buildings with similar or complementary functions)
• Architectural description and features
• Spaces (spatial challenges and restrictions such as heights and areas)
• Existing services and facilities
• Available spaces within the property
• Socioeconomic analysis

## Case Studies of properties in Egypt

### Case study 1: Avierino Building

*Location*

**Figure 6.**
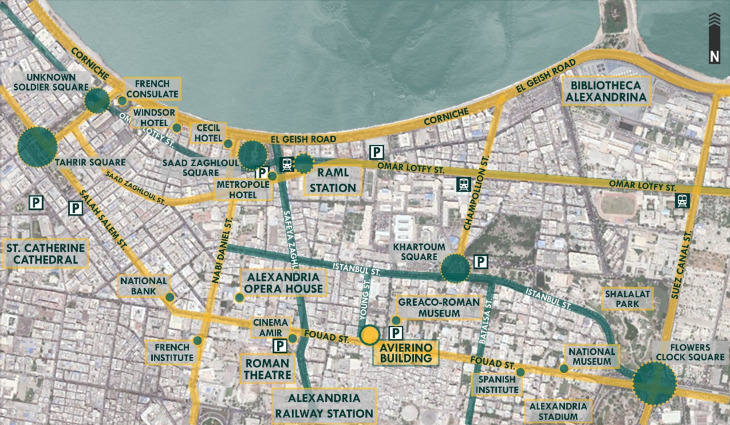
Map of Downtown Alexandria showing the location, main nodes, accessibility routes, parking spaces, and transportation around Avierino Building. Source: Author, 2022.

The Avierino building (AVI) is located in downtown Alexandria, Egypt, along Fouad Street, one of the oldest streets and known to be the oldest functioning street in history, previously named the Canopic Way during the Greek era ( Serageldin, 2002). The building’s location is very important due to its close proximity to various nodes of the city’s most significant functions and activities, as shown in Figure 6; the building is considered to be located in the business district of the city, where major consulates, banks, museums, educational institutions, logistics companies, and sports facilities such as the Alexandria Stadium are located. Also, it is near the Alexandria port, where the majority of the country’s imports are received ( Selim, 2023).

*Accessibility and legibility*

The building is easily accessible *via* diverse methods of private and public transportation, such as personal vehicles, bikes, buses, microbuses, taxis, trams, and trains. The building is within close proximity to Alexandria Railway Station, which provides direct access from all over the city and other cities as well; it is also located near several tram stations and main roads such as El Horeya Street and El Geish, both known to be the main veins of the city where most, if not all, traffic passes through daily ( Selim, 2023). The building has several parking spaces in the surrounding area; these spaces are sufficient to accommodate the building’s users. It is sometimes a bit challenging to find parking spaces during the day due to high traffic, but after rush hour and by night, the area has very low traffic, making it much easier for pedestrians and visitors to explore downtown.

*Surrounding context*

The building is located in the business district in downtown where consulates, banks, companies, museums, educational institutions, cinemas, antique furniture stores, sports facilities, and cultural attractions such as the Alexandria Opera House are all within proximity, this gives the building significant potential as its location attracts high traffic, this traffic includes businessmen and traders because of the logistics companies and the businessmen association headquarters located within the building; other traffic includes tourists and locals visiting surrounding museums such as the Graeco-roman museum and Alexandria National Museum; also students learning languages at the French, Greek, Russian, Spanish, or Italian cultural centers form a significant number of traffic visiting. Finally, bank employees and consulate representatives are also an important sector of the traffic visiting the building ( Awad, 2008; Serageldin, 2002).

*Spaces*

The building has vast heights and spacious areas all over, whether in the ground floor where the heights are up to 6.2 meters high or on the typical floors, which are 4.2 meters high. Since the building is a reinforced concrete structure, it is flexible for opening spaces with each other to make bigger spaces that could be used for diverse functions such as a restaurant or café in any food and beverage project, a co-working space in office projects, or an open space for events, workshops, educational courses, the arts, and cultural projects.

*Architectural description and features*

The building was built in 1928 by the Greek architect Petros Nicolas Griparis after being commissioned by the Avierino family, which was known to have been of Italo-Greek origins. The building was designed in a neo-renaissance style and is an exquisite example showcasing the cosmopolitan spirit of Alexandria; the building had tenants from many countries and locals as well, who all lived together in a socially cohesive environment during that time and till this day ( Awad, 2008).

**Figure 7.**
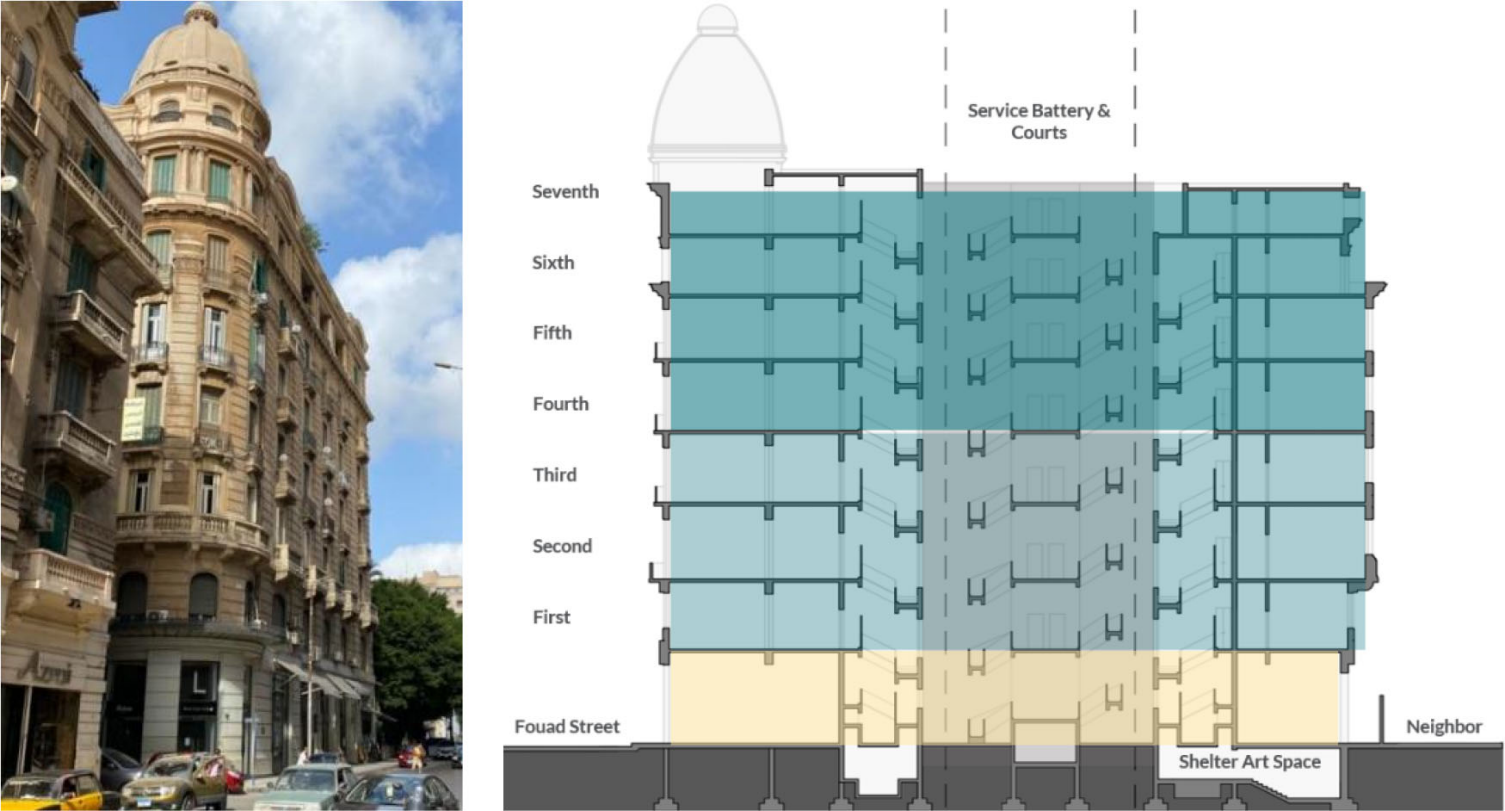
Avierino building from Fouad Street (left) and section showing components (right). Source: Author, 2021.

The building is comprised of a basement, a ground floor, a mezzanine, and seven typical floors, as well as a roof and a dome at the top, as shown in Figure 7. The building has a mixed structural system combining bearing walls and reinforced concrete. Avierino has a built-up area (BUA) of around 11,000 m ^2^ and a floor footprint of 1,350 m ^2^. Each typical floor was originally divided into four apartments, but most of them have been connected to each other through a common corridor that circles around the building’s plan ( Selim, 2023).

*Existing services and facilities*

The ground floor has retail spaces; an art space is in the basement; offices are on different floors; and residential apartments are on the typical floors. It has two main vertical circulation cores, each housing a staircase and an elevator, each in one of the building’s two sides; each side has two apartments, and both sides are connected with a central service core with an elevator; this core is used for services, cleaning, and maintenance uses.

*Available spaces within the property*

**Figure 8.**
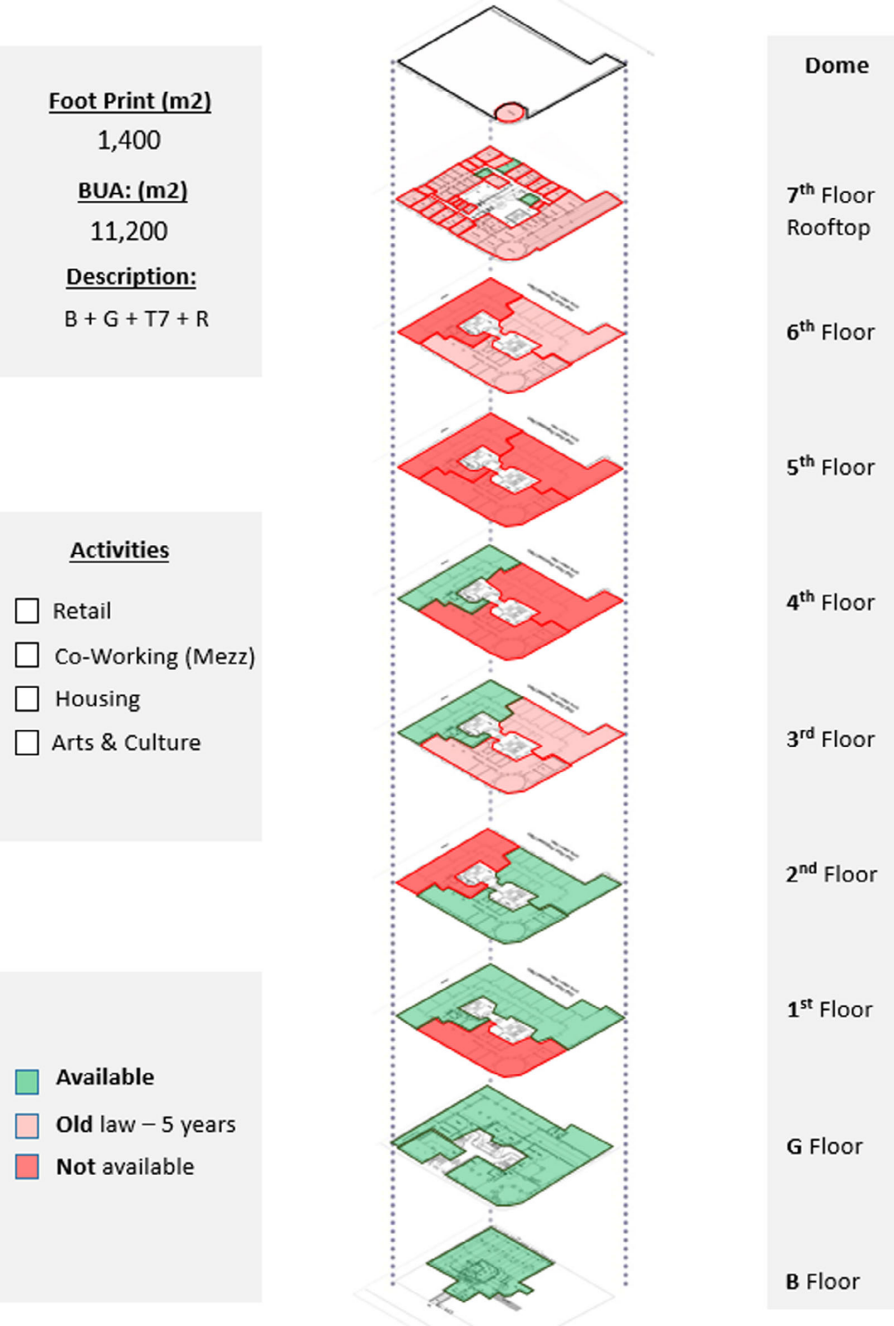
Axonometric diagram of available/unavailable spaces, and potential acquisitions. Source: This figure has been reproduced with permission from Sigma Properties, 2022.

This parameter defines the available spaces in the building in order to prepare a development program for each building and make the vision clear for investors, designers, and other stakeholders regarding the potential investment opportunities and the types of activities that could be incorporated in the building ( Selim, 2023). In Avierino, the basement and ground floor are completely empty, making it a high potential for a commercial, retail, or F&B project. The floors from the first to the fourth all have empty spaces but are not completely empty, so pilot projects such as offices and hospitality could be initiated until more spaces are acquired, as demonstrated in Figure 8.

*Social and economic analysis*

The socio-economic level of the area surrounding Avierino is a mixed one, combining communities from different backgrounds and pay grades in the city, from small to medium entities around the neighboring areas and local businesses and shops, to students and educators from surrounding institutions, all the way to business owners and traders from local antique stores, logistics companies, and banks. The target users and visitors of the building include the local community in downtown, government, banks, and consulates employees and representatives, tourists from foreign countries and other cities in Egypt, and finally, businessmen and merchants visiting the city for commercial purposes ( Said, 2016).

*Development model*

**Figure 9.**
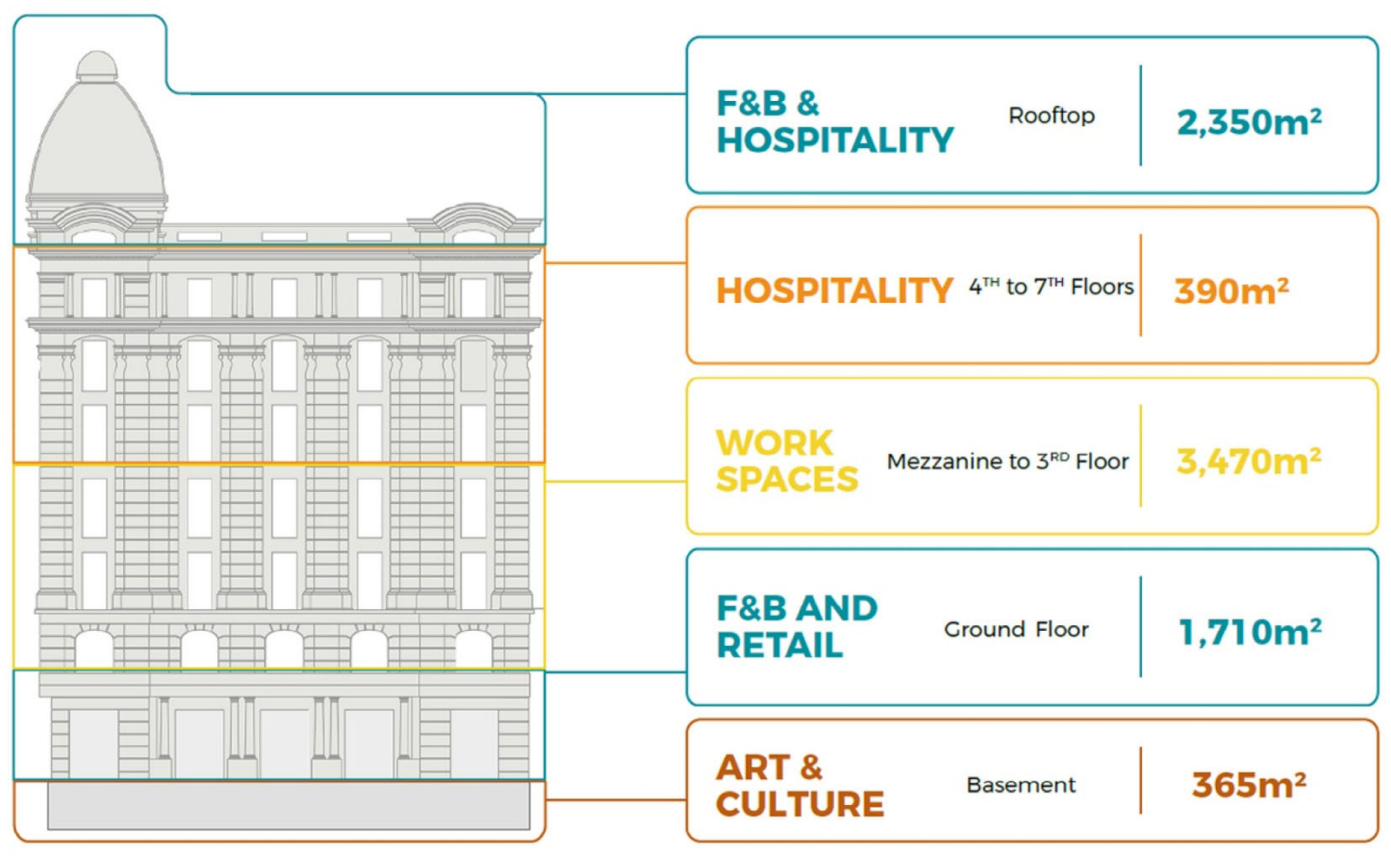
Proposed development model for future vision based on the data demonstrated in the axonometric diagram ( Figure 8). Source: This figure has been reproduced with permission from Sigma Properties, 2022.

After the detailed analysis of the Avierino building and understanding its location, accessibility, surrounding context, spatial characteristics, architectural features, existing service, availability of spaces, and analyzing the socio-economic status of the area and the local community, the program developers, along with the investors and other stakeholders, formulate a general vision regarding the highest and best use. The analytical process that precedes the decision regarding the most suitable function and component mix is what leads the involved stakeholders to develop a program in which all future projects are going to follow; this program also suits the existing functions and uses already existing in the property and surrounding properties as well, as shown in Figure 9.

The development model shown in Figure 9, incorporates the following functions: F&B and retail spaces in the ground floor and rooftop; offices and workspaces in the mezzanine floor up to the third floor; hospitality services in the fourth floor up to the seventh floor; and an art space as an arts and culture function in the basement.

**Figure 10.**
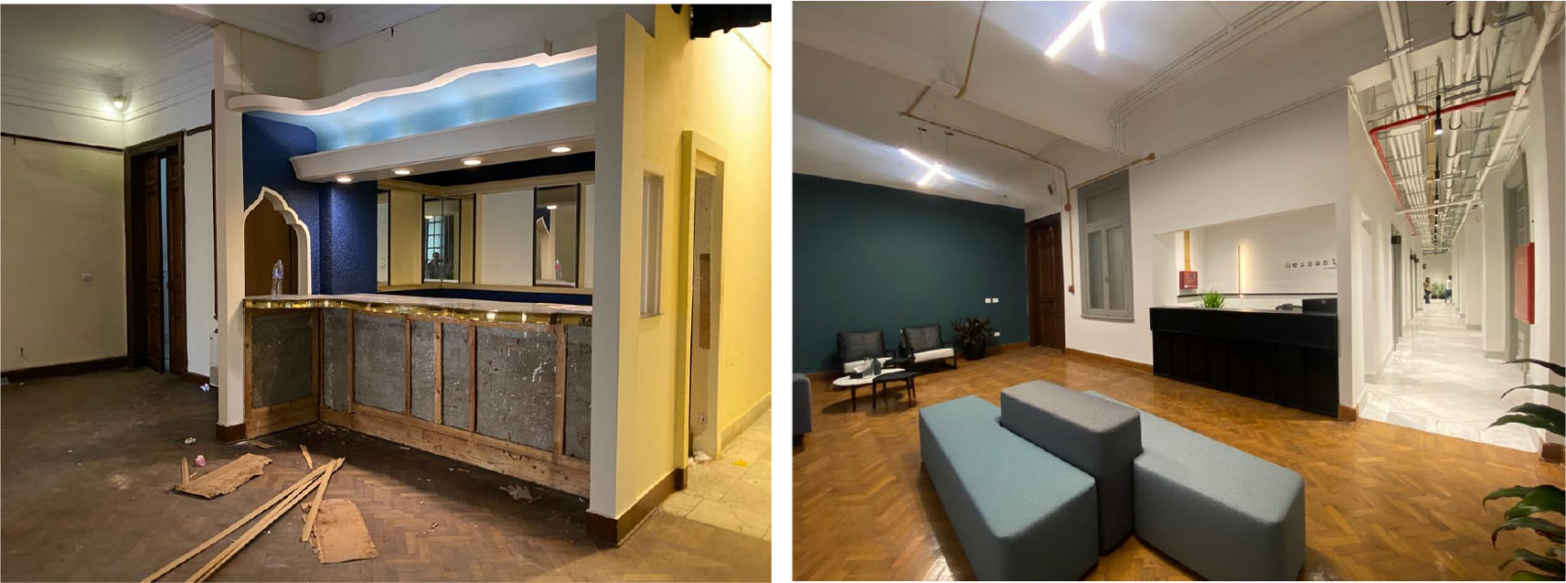
Before and after status of the reception area of the project, the original parquet floors were restored and the existing features where maintained. Electrical systems were installed in an exposed manner in order to preserve the vast heights of the interior spaces. Source: This figure has been reproduced with permission from Sigma Properties, 2022.

**Figure 11.**
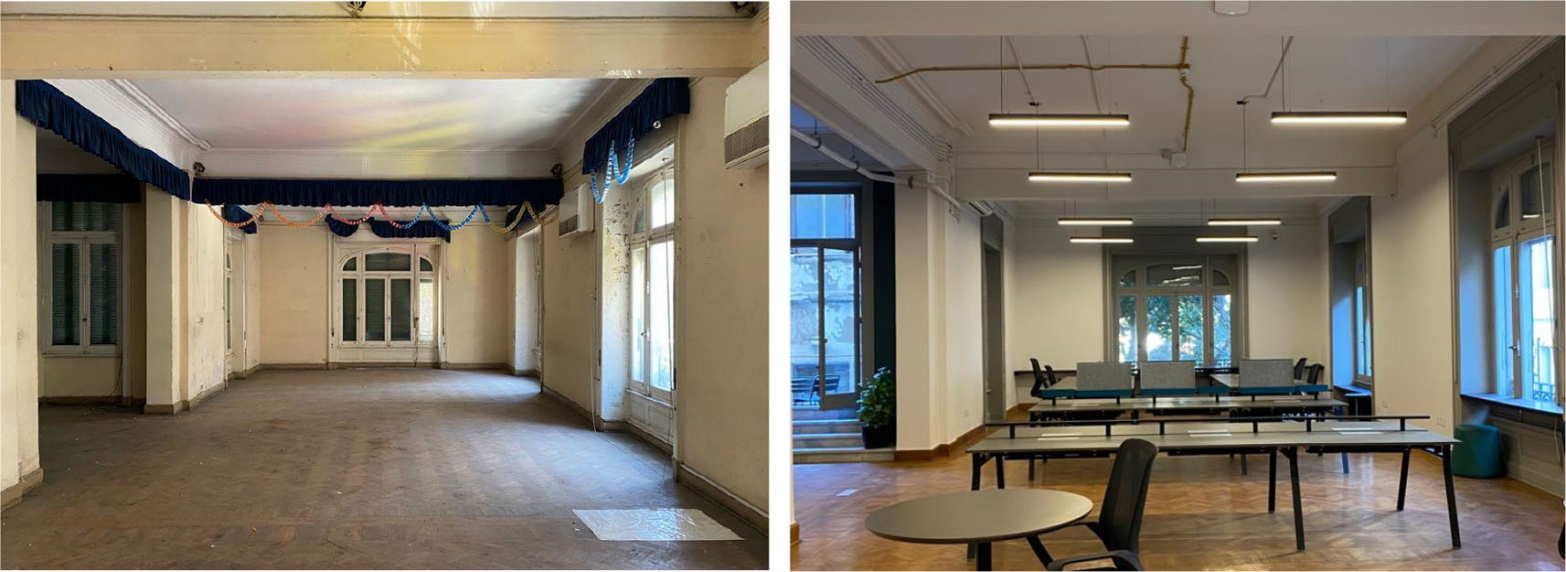
Before and after status of what currently is a Coworking space which was originally an open area used for educational purposes, also an outdoor terrace was introduced and access was created by the steps added through a window in order to maintain the integrity of the building’s structure and original openings. Source: This figure has been reproduced with permission from Sigma Properties, 2022.

**Figure 12.**
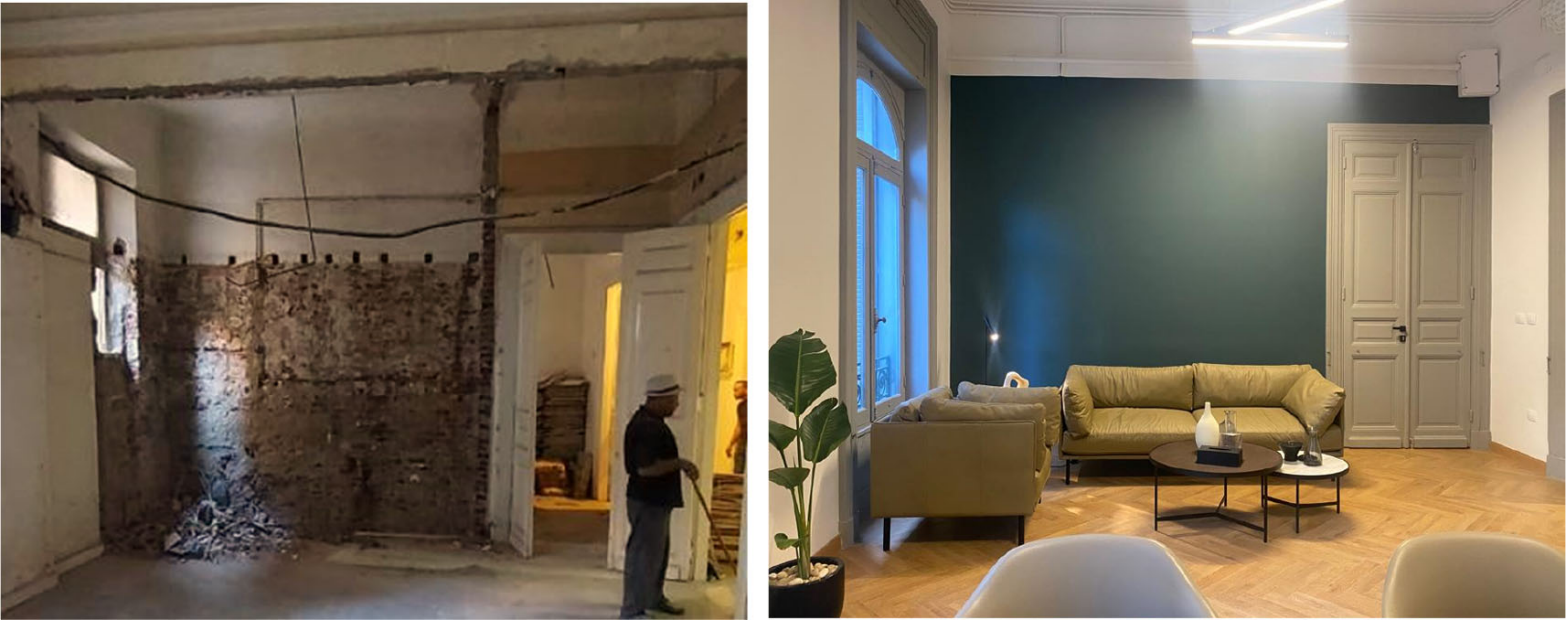
Before and after photos of what currently is a meeting room; the previous tenant destroyed the original window and created a wall in its place. Transgressions were removed and a new window replicating the original design was added once again. Source: This figure has been reproduced with permission from Sigma Properties, 2022.

‘Mezzanine Offices and Coworking Spaces’ demonstrated in Figures 10- 12, is an example of one of the main uses that have been introduced in the Avierino building as a pilot project to incorporate the function of offices and workspaces and is intended to be one of the brands that could potentially be replicated in other buildings as part of the adaptive reuse strategy ( Selim, 2023).

### Case study 2: Little Venice Building

*Location*

**Figure 13.**
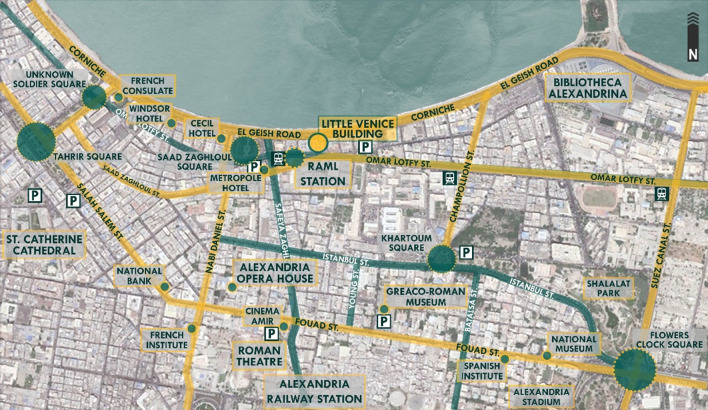
Map of Downtown Alexandria showing the location, main nodes, accessibility routes, parking spaces, and transportation around Little Venice building. Source: Author, 2022.

The Little Venice (LV) building is located in a prime waterfront location in downtown Alexandria along El Geish Road; it is the most important and most famous road in Alexandria since it covers the majority of the Mediterranean city’s waterfront and is a critical traffic vein where all types of light transportation methods exist and all of the city’s development and urban sprawl are condensed around due to the road’s importance and its sentimental connection with all citizens of Alexandria, as shown in Figure 13. The building is considered to be located in the historical district of the city, where ancient temples of Egypt previously existed, such as Cleopatra’s Temple, and ancient sites of Roman Egypt. Also, it is near the Alexandria port, where the majority of the country’s imports are received ( Serageldin, 2002). The building has a direct view of the waterfront, an area famously known as the Eastern Harbor where *Portus Magnus,* one of the oldest ports in the world, was built and is now an archeological site with ancient underwater remains of Alexander the Great’s royal fleet ( Selim, 2023).

*Accessibility and legibility*

The building is easily accessible via diverse methods of private and public transportation, such as personal vehicles, bikes, buses, microbuses, taxis, trams, and trains. The building is directly located from the front elevation on El Geish Road, and from the opposite side, it is directly facing Raml Station, the first tram station on the main line of Alexandria’s tramway ( Selim, 2023). The building has several parking facilities in the surrounding areas within walking distance, with sufficient spaces making it easy to find parking spots at any time of the day except for rush hour, as is the case in downtown in general.

*Surrounding context*

The building is located in a district that could be described as a historical, recreational, commercial, and touristic district in downtown where public squares, hotels, cinemas, consulates, banks, and major cultural and touristic attractions such as the Alexandria Library famously known as the Bibliotheca Alexandrina, are all within proximity; this gives the building significant potential as its location attracts high traffic, this traffic includes tourists and locals visiting the Alexandria Library for attending cultural events or for educational purposes, or staying for touristic purposes in general looking to stay in a prime waterfront location; also, employees or businessmen visiting the city for business meetings and commerce, or locals visiting for convenient shopping, dining, or going to cinemas and theaters in the area ( Serageldin, 2002).

*Spaces*

The building has considerably high ceilings but limited areas due to structural limitations caused by the building’s design, except for the ground floor, which has open areas and ceilings up to 5.2 meters high. The typical floors are 4.2 meters high, and since the building is a reinforced concrete structure, it is flexible for opening spaces with each other but with certain limitations in order to maintain the building’s structural and architectural integrity.

*Architectural description and features*

The building was built in 1929 with a unique architectural style combining Neo-Gothic and Venetian elements influenced by the local architecture of Alexandria. It was built by Giacomo Alessandro Loria, an architect born in El Mansourah to a Jewish family from Livorno that settled in Egypt during the time of Mohammed Ali. The building had an exquisite exterior design, making it the best façade of the year in 1929. The building had beautiful mosaic medallions and tiles on its corner towers, each with a unique design ( Selim, 2023); it also had Moorish arches with Gothic detailing resembling those of the Palazzo Ducale in Venice, Italy ( Awad, 2008).

**Figure 14.**
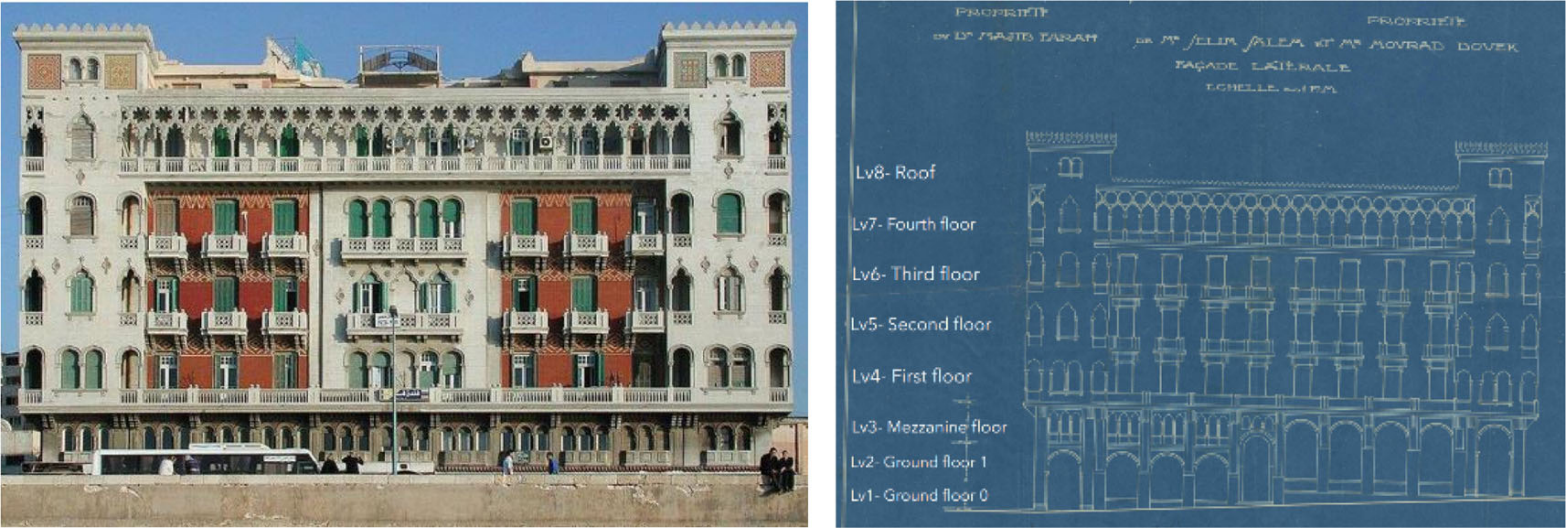
Exterior design of main façade (left), original façade blueprint (right). Source: This figure has been reproduced with permission from Sigma Properties, 2022.

The building is comprised of a ground floor that is divided on two levels due to the ground’s slope, a mezzanine, and four typical floors, as well as a roof, as shown in Figure 14. The building has a mixed structural system combining bearing walls and reinforced concrete. It has a BUA of around 5,800 m ^2^, and each typical floor was originally divided into four apartments.

*Existing services and facilities*

The building has empty retail spaces on the ground floor, various offices and clinics on different floors, and a hotel on the first and second floors. It has one main vertical circulation core housing a staircase and an elevator and two courts; each court has a service core supplied with a staircase; this core is used for services, cleaning, and maintenance uses.

*Available spaces within the property*

**Figure 15.**
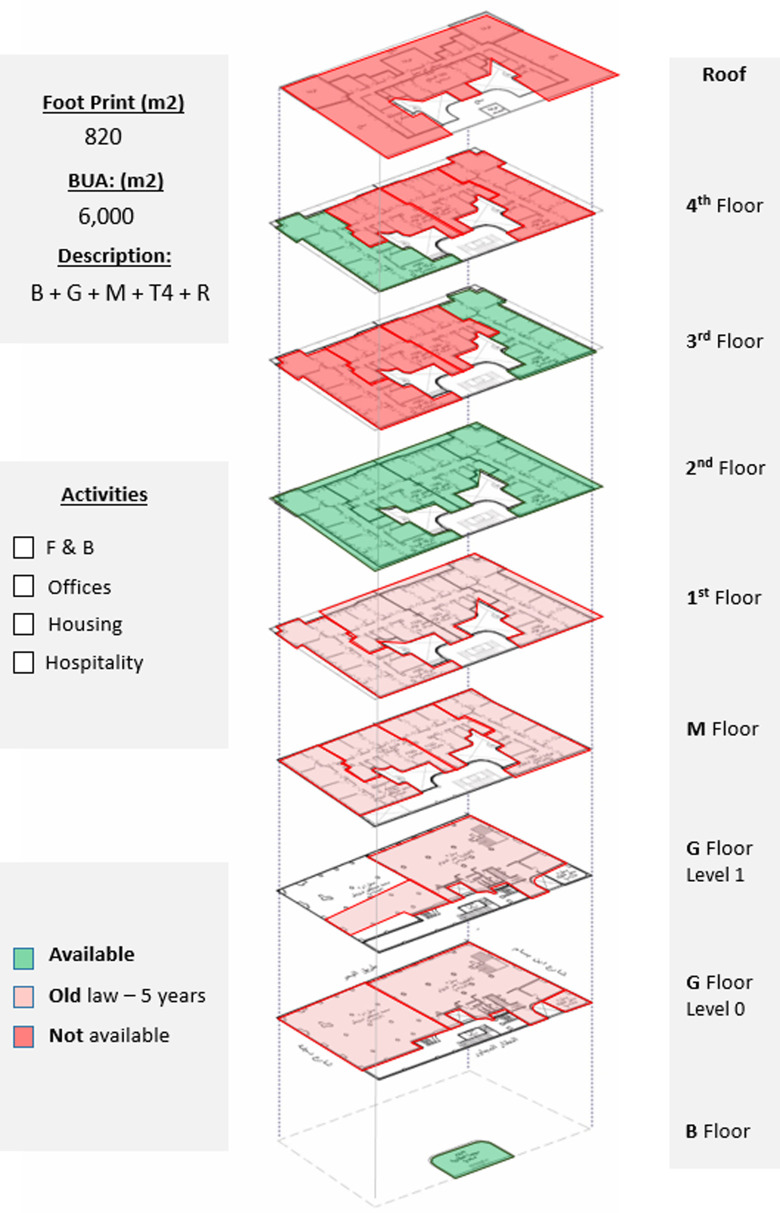
Axonometric diagram of available, unavailable, and potential acquisitions. Source: This figure has been reproduced with permission from Sigma Properties, 2022.

The ground floor and mezzanine are currently unavailable due to ‘old law’ lease contracts, and from the typical floors, only the second floor is fully available, which could be developed as a hotel due to the building’s prime location and previously existing functions and permits ( Selim, 2023); also, a few apartments are available on the third and fourth floors, which could also be developed as part of a hotel project or other hospitality services, as demonstrated in Figure 15.

*Social and economic analysis*

The area surrounding the Little Venice building is similar to Avierino since they are within proximity of each other, meaning that it combines communities from different backgrounds and levels as well. The only difference is that Little Venice’s prime waterfront location gives it a significantly higher potential to attract visitors with a higher economic level and purchasing power in general, so a bigger percentage of hospitality services is required, preferably a boutique hotel, and a higher category of finishes, furniture, materials, and equipment will be required ( Said, 2016).

*Development model*

**Figure 16.**
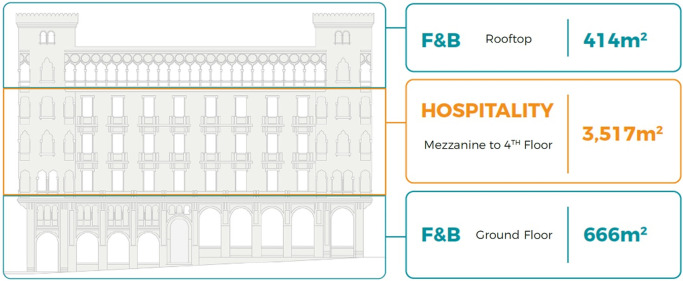
Proposed development model for future vision based on the data demonstrated in the axonometric diagram ( Figure 15). Source: This figure has been reproduced with permission from Sigma Properties, 2022.

After the detailed analysis of the Little Venice Building and understanding its location, accessibility, surrounding context, spatial characteristics, architectural features, existing service, availability of spaces, and analyzing the socio-economic status of the area and the local community, the program developers, along with the investors and other stakeholders, formulate a general vision regarding the highest and best use, as shown in Figure 16.

The development model incorporates the following functions: F&B on the ground floor and rooftop, and premium hospitality services on the mezzanine floor up to the fourth floor, due to the prime waterfront location of the building.

**Figure 17.**
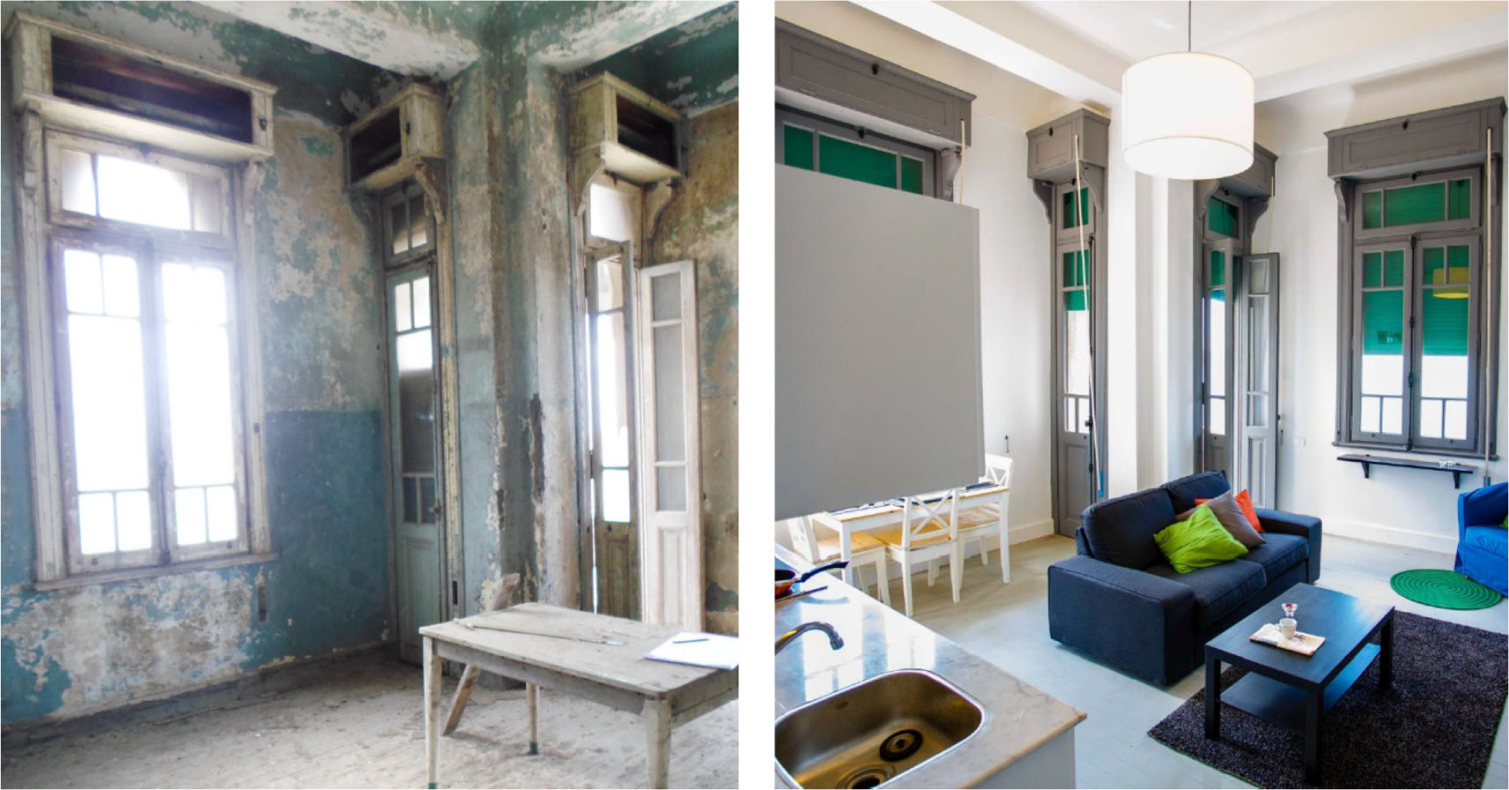
Before and after status shows how the existing elements were preserved such as the wooden doors and windows, and the original roller shutters in order to preserve the building’s identity and unique heritage significance. Source: This figure has been reproduced with permission from Sigma Properties, 2022.

The ‘Venetian Suites’ project demonstrated in Figure 17, is an example of one of the main uses that have been introduced in the Little Venice building as a pilot project to incorporate the function of hospitality services and, following its substantial success considering the financial aspects as well as the users’ feedback, it is now part of a larger scale hospitality project that could potentially be replicated in other buildings as part of the adaptive reuse strategy ( Selim, 2023).

### Case study 3: Ouzonnian Building

*Location*

**Figure 18.**
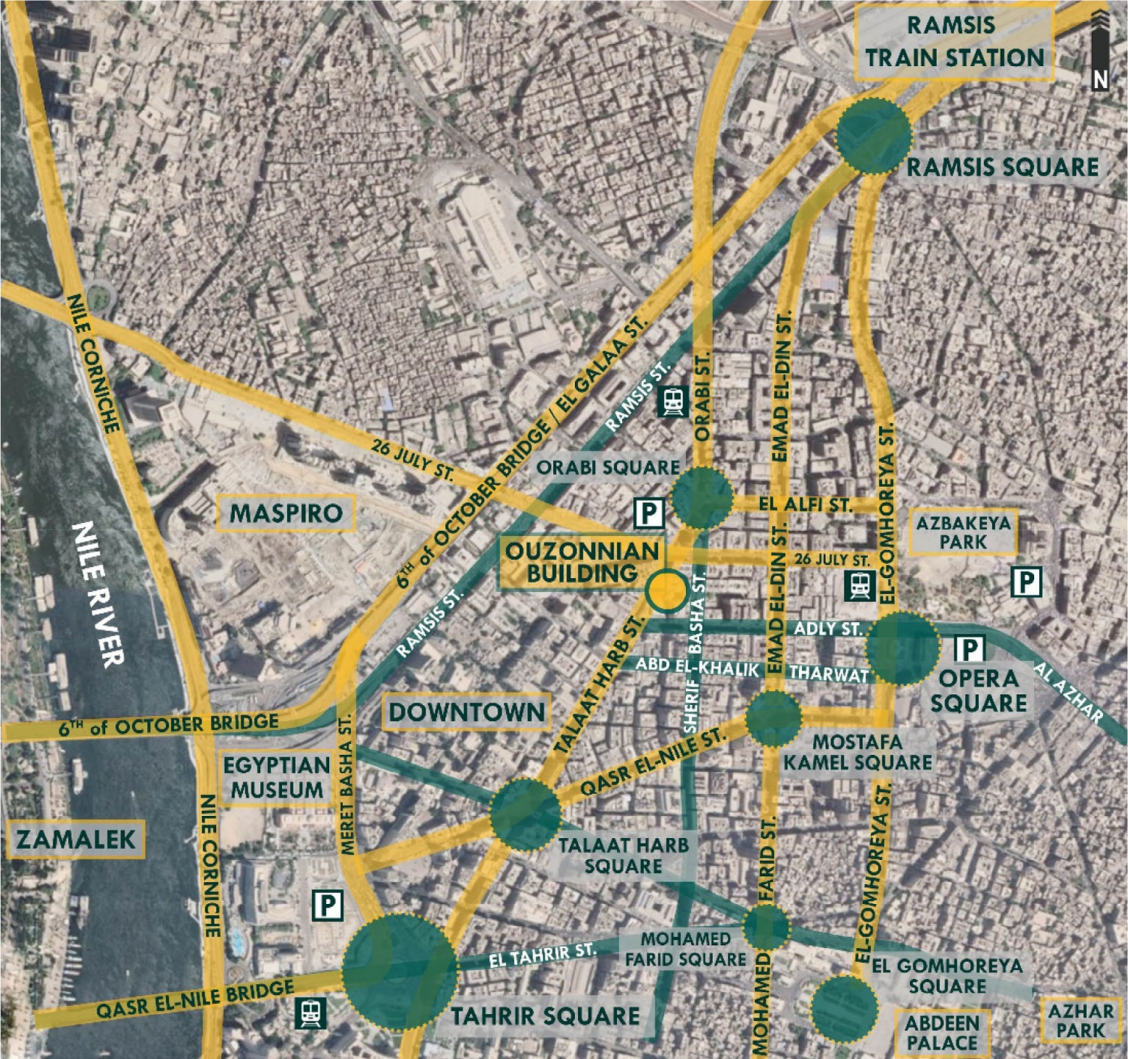
Map of Downtown Cairo showing the location, main nodes, accessibility routes, parking spaces, and transportation around Ouzonnian building. Source: Author, 2022.

Located in downtown Cairo, along Talaat Harb Street, which was previously named Suleiman Pasha Street during the time of Mohammed Ali and renamed in 1954. It is one of the most important streets in downtown Cairo and is considered the main vein connecting Tahrir Square and Talaat Harb Square, as shown in Figure 18. The street was a hub for activities and social interaction among Cairo’s upper class and foreign European communities. The influence of European culture and architecture is still evident and embodied in French neoclassical architecture ( Selim, 2023).

*Accessibility and legibility*

The building is in a prime location considering accessibility, knowing that it is connected directly to the Qasr El Nile Bridge, the 6th of October Bridge, and the Azhar Tunnel and Bridge, connecting downtown to major areas in Cairo. The building’s location is easily accessible via different methods of transportation and through various alternative routes. The building is located within close proximity to metro stations and Ramsis railway station, the most important station to visitors from all around Egypt as the connecting railway trains all stop at Ramsis station and visitors then use other methods of transportation to reach their destinations around Cairo ( Selim, 2023). Parking facilities are available directly next to the building, as well as in the surrounding areas such as the Opera garage and the Tahrir garage.

*Surrounding context*

The building is considered to be in a commercial area, as locals and the majority of traffic visiting the street visit to shop for conveniently priced clothing and other accessories. Other visitors are employees working with governmental institutions, banks, tourism companies, etc. Also due to the building’s close proximity to Tahrir Square, where visitors from all over Egypt and tourists meet, either as a station to commute to and from their cities or visit the Egyptian Museum and other touristic locations. The building is situated next to consulates and multiple governmental institutions; also surrounding the building are hotels, banks, retail stores, restaurants, cinemas, bookstores, offices, educational institutions such as the Greek campus and the American University in Cairo campus, sporting clubs, and cultural magnets such as the Cairo Opera House. Furthermore, the Nile Corniche and tourist attractions such as the Egyptian Museum, Abdeen Palace, Cairo Tower, and many other locations are close to the building ( Mubarak, 1989).

*Spaces*

The Ouzonnian building has a relatively small floor footprint, meaning that its areas will be limited considering functions that require open or large spaces; also, the floors have limited heights due to its modern construction system, unlike Avierino and Little Venice. The ground floor height is up to 4.5 meters, and the typical floors are 3.2 meters high. The building has lower heights, smaller courts, and minimal features; this has an impact on technical and design-related decisions.

*Architectural description and features*

This building was built by the Egyptian architect Sayed Karim, born in 1911 in Quesna, Egypt. He was a professor of architecture at Cairo University, and he registered the first consulting office in Egypt for architecture and planning in 1939; he also issued the first architecture and arts magazine in Egypt in the same year ( Selim, 2023).

**Figure 19.**
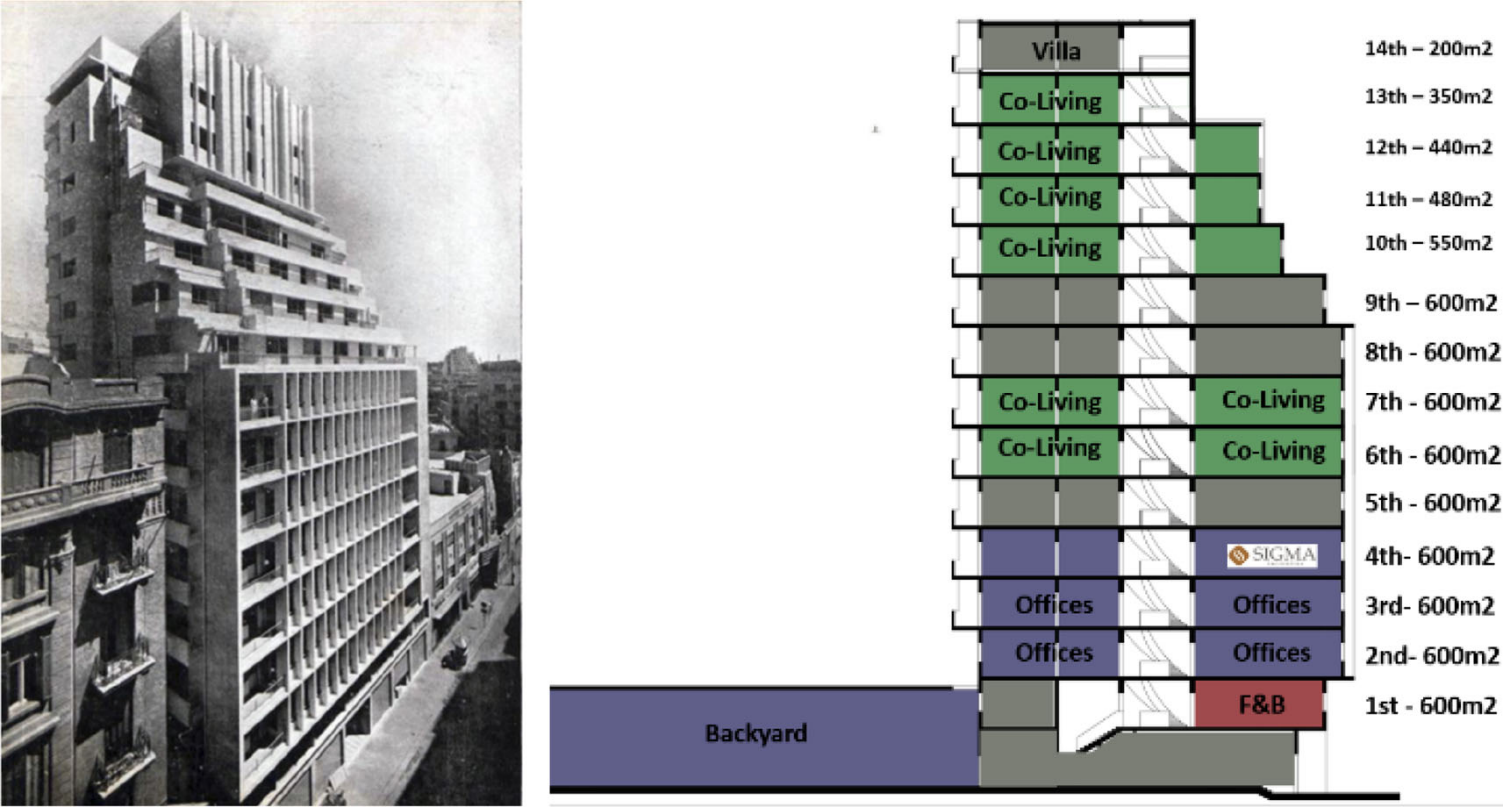
Historical photo of the building (left), section showing components and spaces. Source: This figure has been reproduced with permission from Sigma Properties, 2022.

The building was recognized as one of the first modern buildings in Egypt and the tallest building in Cairo at that time. It has a reinforced concrete structure system and it is comprised of a ground floor that is connected to a spacious backyard, a mezzanine, seven typical floors, four upper floors with different areas that become smaller every floor, and two final floors with even smaller areas as shown in the building’s images. It has a BUA of around 8,200 m ^2^, each typical floor was originally divided to four apartments but some of them have been connected to each other, as demonstrated in Figure 19.

*Existing services and facilities*

The building was designed to host various functions, with the ground floor containing commercial spaces. The first floor had a restaurant, a dancing hall, and a rotunda that featured an open-air garden. The seven typical floors functioned as apartments and offices, the four upper floors featured a hotel with a private garden, and the top floors housed residential units. Nowadays, the building has retail stores on the ground floor, offices on the mezzanine and first floors, and residential and studio apartments on the typical floors. The building has a central vertical circulation core in its center with three elevators and a staircase, and it has two courts, both of which have a staircase, but only one of them has a service elevator; these service courts were used for services, cleaning, and maintenance ( Selim, 2023).

*Available spaces within the property*

**Figure 20.**
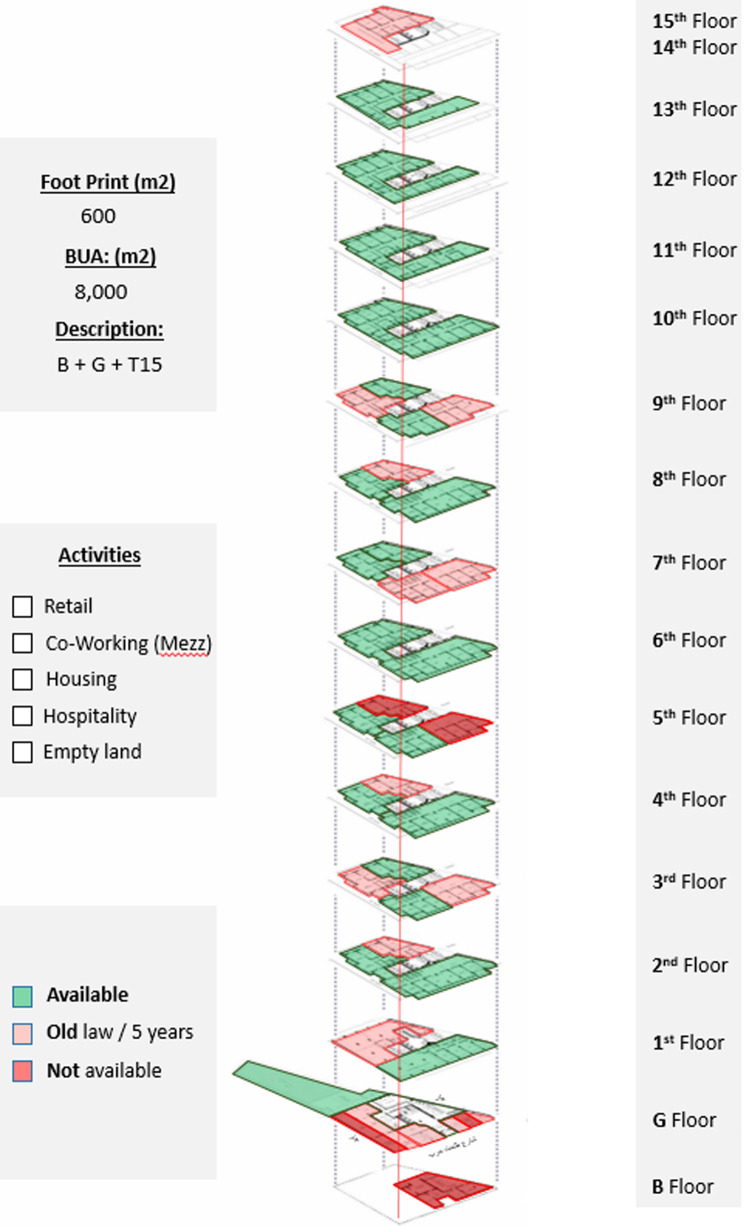
Axonometric diagram of available, unavailable, and potential acquisitions. Source: This figure has been reproduced with permission from Sigma Properties, 2022.

The ground floor has retail stores with old contract rentals; the backyard, on the other hand, is acquired and could be developed as a F&B project; the floors from the first till the fourth are all office spaces; the majority of the spaces are acquired and could be developed as a part of a project. The fifth, seventh, and eighth floors are also not fully acquired, but each floor has available spaces; all are residential spaces ( Selim, 2023). The sixth floor is fully available and has been utilized as a pilot for an AirBnB ( https://www.airbnb.co.uk) hospitality project that is currently undergoing the extension phase on the tenth through the thirteenth floors, as demonstrated in Figure 20.

*Social and economic analysis*

The area surrounding Ouzonnian is similar to those of Avierino and Little Venice considering their locations in downtown areas, but the fact that Ouzonnian is in Cairo significantly changes the equation, knowing that the traffic in Cairo is much higher, especially tourists, backpackers, artists, and entrepreneurs working on the go. This gives Ouzonnian a higher success rate when considering mixed-use developments in general due to the feasibility of the projects in this area. The expected target users are mostly younger generations of entrepreneurs and travelers looking for a local experience and a conveniently priced stay such as AirBnB and studio apartments. Also, such users would require places to work from, such as private offices and co-working spaces that would be complimented with F&B services to complete the component mix in the building, making it an independent, self-sufficient destination.

*Development model*

**Figure 21.**
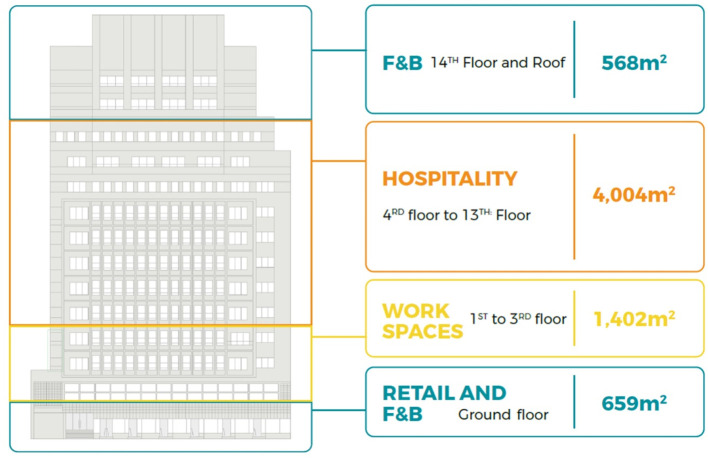
Proposed development model for future vision based on the data demonstrated in the axonometric diagram ( Figure 20). Source: This figure has been reproduced with permission from Sigma Properties, 2022.

After the detailed analysis of the Ouzonnian Building and understanding the parameters of the property assessment, the program developers, along with the investors and other stakeholders, formulate a general vision regarding the highest and best use, as shown in Figure 21.

The development model incorporates the following functions: F&B and retail spaces on the ground floor, fourteenth floor, and rooftop; offices and workspaces on the first floor up to the third floor; and hospitality services on the fourth floor up to the thirteenth floor.

**Figure 22.**
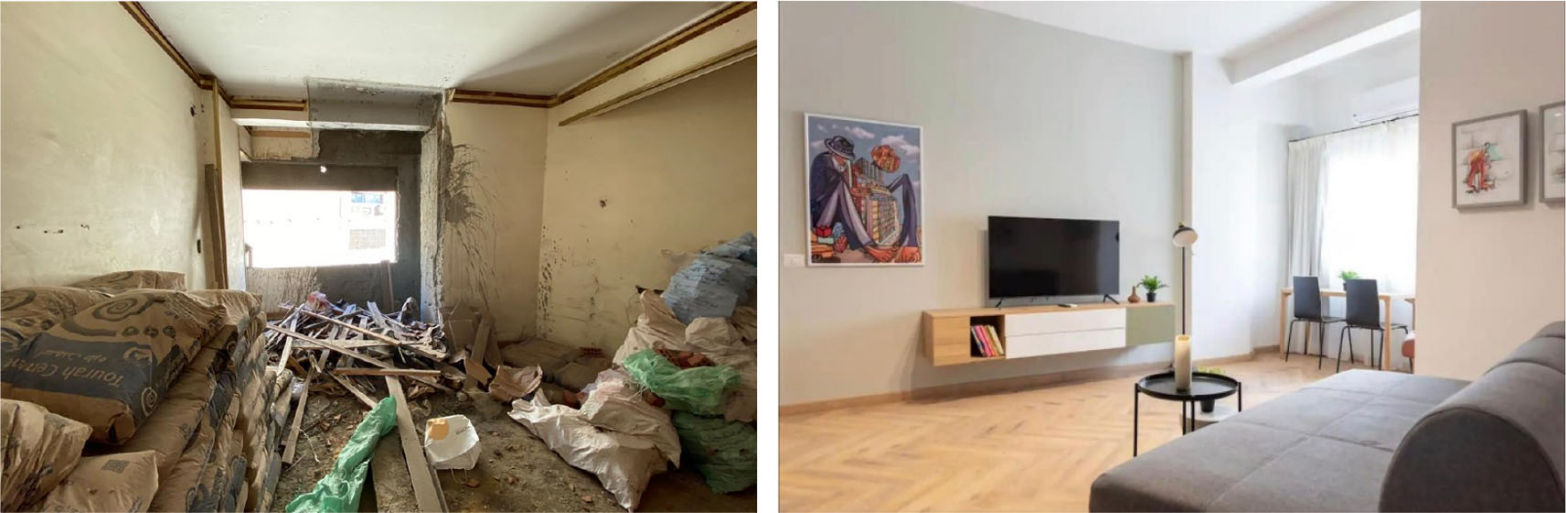
Before and after photos showing how the openings and windows were restored to their original size and look to maintain the harmony of the building’s façade. Source: This figure has been reproduced with permission from Sigma Properties, 2022.

**Figure 23.**
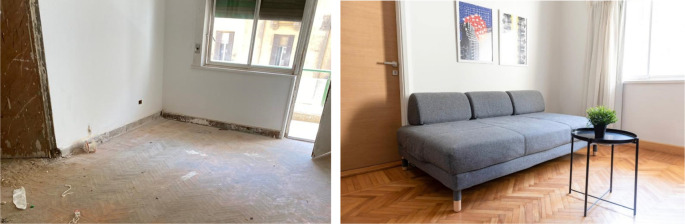
Before and after status of one of the units which had original parquet floors, those were restored and reutilized in the space, also another feature that was restored was the original windows and roller shutters. Source: This figure has been reproduced with permission from Sigma Properties, 2022.

**Figure 24.**
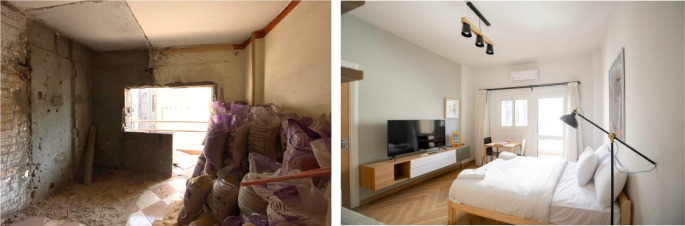
The photos above demonstrate the before and after status of one of the studios in the project. Source: This figure has been reproduced with permission from Sigma Properties, 2022.

‘Grey Studio Apartments’ demonstrated in Figures 22- 24, is an example of one of the main uses that have been introduced in the Ouzonnian building as a pilot project to incorporate the function of hospitality services that resemble AirBnB stays and, following its substantial success considering the financial aspects as well as the users’ feedback, it is now part of a larger scale hospitality concept that could potentially be replicated in other buildings as part of the adaptive reuse strategy ( Selim, 2023).

## Results

The following table summarizes the results derived from the previous analyses, as the author selected three buildings in downtown areas of Alexandria and Cairo to be part of the comparative analysis approach, which was performed with the aim of producing and assessing decision-making parameters that assist developers and other stakeholders involved in the adaptive reuse of heritage buildings by acting as guidelines that those stakeholders could potentially utilize for evaluating heritage properties and reaching an expert decision regarding the highest and best use of the spaces provided in those assets. The table also provides a brief description of the evaluation of those parameters in order to complete the comparative analysis.

**Table 5.** Comparative analysis of decision-making parameters of the case studies in brief. Source: Author, 2022.

Parameter	Location	Accessibility and Parking	Surrounding Context	Spatial Restrictions	Arch. Features	Facilities	Available Spaces	Socio-Economic Status
Avierino	Prime Business District	Easily accessible, multiple parking spaces	Commercial, cultural hubs, educational institutions	High ceilings and potential for open spaces	Unique ornaments and arch. design	Elevators, services in good condition	Retail available but limited acquisition	Average socio-economic levels
Little Venice	Prime Waterfront Location	Average accessibility, limited parking	Commercial area, hotels & consulates	High ceilings but smaller, limited areas	Unique ornaments and arch. design	Limited services and facilities	Limited spaces for development	High due to target users (prime location)
Ouzonnian	Touristic location, high traffic	Average accessibility, limited parking	Central hub, but noisy, and street vendors	Low ceilings, limited areas but flexible RC system	Minimal design, low features	Average services, need improving	Retail not available but most spaces acquired	Average socio-economic levels

Level of parameter: ● High ● Average ● Low.

## Discussion

**Figure 25.**
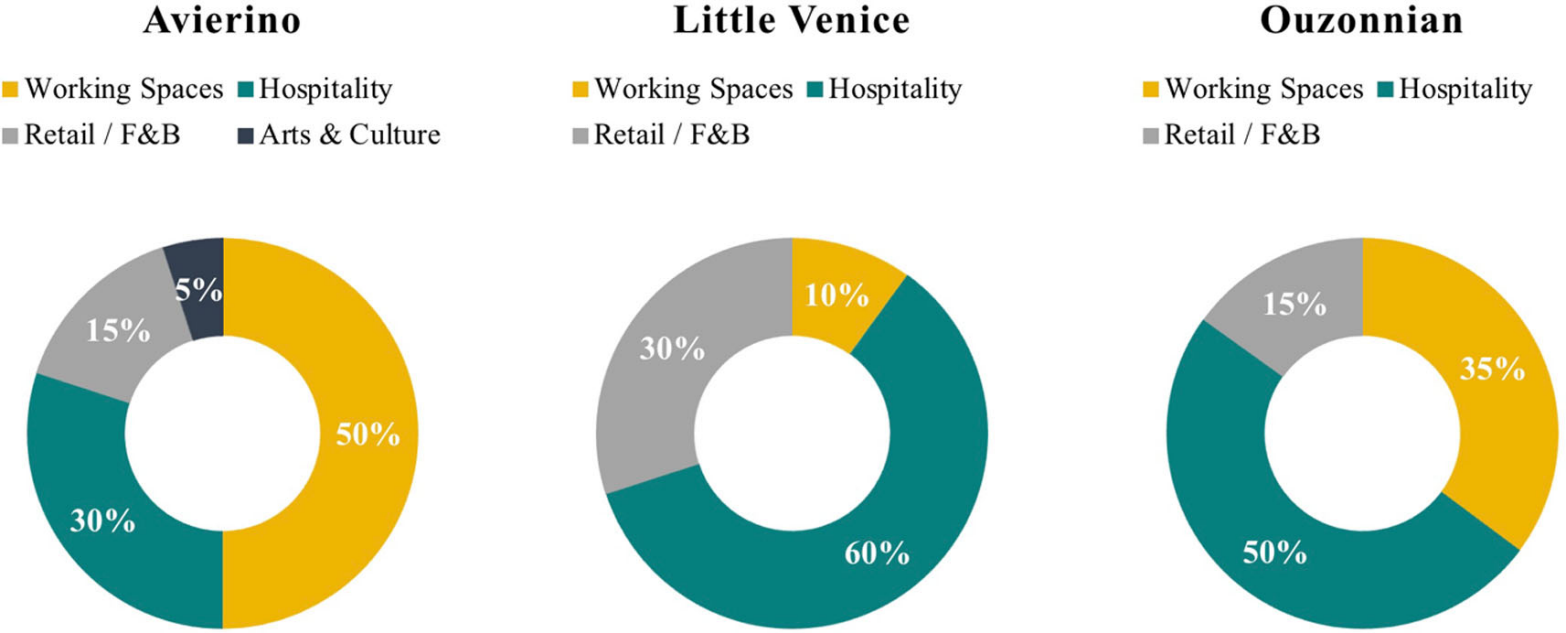
Pie charts showing the proposed development program for each building based on the decision-making parameters. Notes: F&B – food and beverage. Source: Author, 2022.

The following Figure 25 shows the proposed business model for each of the case studies analyzed throughout this research, considering the available spaces and the spaces to be available within a five-year development pipeline. It is evident that the optimum component mix and percentage distribution differ in each property, and in each case, there is a recommended percentage for the main functions as well as secondary ones.

When it comes to the functions forming the component mix in the adaptive reuse of heritage buildings, it is evident that there are a number of common functions that are constant and are replicated throughout other developments. These functions are: retail and commercial, F&B, hospitality, offices and co-working space, educational facilities, and arts and culture. Some of those functions have become of critical significance following the pandemic and international conflicts, while others have always been a permanent component of any property ( NSW Department of Planning and RAIA, 2008).

The outcome of this research shows how these functions are supposed to coexist and be coherent with each other to make a successful adaptive reuse development program, but what is clear is that those functions are not the same in the percentage of their incorporation in the development, but they are variable in scale and category depending on the decision-making parameters ( Elsorady, 2020); in one of the cases, the building is a waterfront property in a prime touristic location, so the focus on hospitality is higher and it gets a bigger scale in the building and a higher category considering the level of finishing, furniture, equipment, and overall technology utilized in that project ( Willson and Mcintosh, 2007). In other cases, the building could be in a Commercial Business District where existing traffic require F&B services as well as offices and Co-working spaces, in that building the scale of those functions are bigger and more significant, however, this doesn’t mean that hospitality services won’t be provided.

Also, another adaptive reuse (AR) scenario is when a building is fully available or the majority of its spaces are acquired, and within legible, connected spaces, it has a lot of potential to be developed as a whole and operated as well; this is a common scenario where projects are usually limited to parts of a building, not the building as a whole, since most buildings in downtown have residents, and also because that is a relatively smaller investment considering the acquisition of whole buildings with 100% empty spaces.

Furthermore, for projects located in prime areas with prime views, it is recommended to develop a program with higher percentages of hospitality functions. For hospitality functions in an area where most traffic and locals are of a lower socio-economic level, AirBnB, hostels, and studio apartments are recommended to suit the needs and capabilities of the area. On the other hand, in areas with target users with a higher level of income and economic capabilities and a highly touristic presence, it is recommended to provide the users with premium hospitality services such as hotels, boutique hotels, serviced apartments, or studio apartments as well, but with a higher grade of design considering finishes, materials, and furniture ( Freund de Klumbis and Munsters, 2005).

Table 6 demonstrates the implementation of the evaluation system which was developed by the researcher in order to perform an overall grading for the three buildings based on the sub-parameters to achieve a detailed evaluation.

**Table 6.** Table demonstrates the application of the detailed evaluation of the three buildings based on the developed impact factors. Source: Author, 2023.

Main Parameter	Evaluation based on sub-parameters Avierino Building (AVI), Little Venice (LV), and Ouzonnian (OUZ)	Grade (Low = 0, Average = 1, High = 2)		
		AVI	LV	OUZ
Location	Views	1	2	1
	Historical Significance (Location Attributes) (Touristic or commercial)	2	2	2
	Minimum Environmental Impact (Noise or other pollution level)	1	0	0
	Market Demand	1	2	1
Total grade:		5/8	6/8	4/8
Accessibility	Diverse Modes of Transport (Private cars and vehicles)	2	2	2
	Connected to Main Roads	2	2	2
	Public Transport (Train, Tram, Metro, Buses, Taxis, etc.)	1	2	2
	Proximity of Parking and Facilities	2	1	1
Total grade:		7/8	7/8	7/8
Surrounding context	Proximity to Amenities	2	2	2
	Safety	2	2	2
	Market Value	1	2	1
	Engagement with Surrounding Community (Vitality)	2	1	2
Total grade:		7/8	7/8	7/8
Architectural description	Building Condition	2	2	2
	Historical Significance (Architect & Building)	2	2	2
	Overall Features & Ornaments	2	2	0
	Façades State & Features (Minimum Transgressions)	1	2	1
	Old Elements of Interior Spaces still intact (Floors, Windows, etc.)	2	1	1
	Category of Building (Heritage listing, building is protected and flexible for development)	1	2	1
Total grade:		10/12	11/12	7/12
Spatial characteristics	Structure System (Type, state, and flexibility of opening spaces)	2	1	2
	Open Spaces Availability	2	0	2
	Heights	2	2	1
	Sizes of Openings (Windows of Facades and Courts)	2	1	2
Total grade:		8/8	4/8	7/8
Facilities	State and Scale of Available Infrastructure (MEP)	1	1	0
	Elevators and Service Cores	2	1	1
	State and Scale of Courts	1	1	0
	Roof and Basement availability and state	2	0	2
Total grade:		6/8	3/8	3/8
Available spaces	Legal Situation (Percentage of Available spaces and types, old or new law rentals)	1	1	2
	Ground Floor Area & Availability for Retail/Commercial uses	2	1	1
	Roof Availability and Readiness for Development	1	0	2
	Basement Availability and Readiness for Development	2	0	2
	Connectivity of Spaces	1	1	1
Total grade:		7/10	3/10	8/10
Socioeconomic analysis	Economic Conditions of local community	1	1	1
	Cultural Level	2	1	1
	Social Level	1	1	1
	Job Growth (Increasing Level of Job Opportunities)	1	0	1
	Population levels	1	1	2
Total grade:		6/10	4/10	6/10
Total overall grade:		56/72 **(78%)**	45/72 **(62.5%)**	49/72 **(68%)**

This evaluation is performed on the three buildings of Avierino, Little Venice, and Ouzonnian and shows the differences between them considering each parameter. The total evaluation of each building represents its potential in the AR industry, considering the development model, the building’s historical value and significance, its architectural and structural state, the availability of spaces to be utilized, as well as the building’s degree of ability and flexibility in housing different functions and therefore becoming a hub that serves the needs of the users and local community.

In the case of the Avierino building, it has the highest overall grade since the building has an overall high grade considering all of the parameters; on the other hand, the Little Venice building has the lowest grade of the three buildings, not because it has less historical significance or poor architectural features, but because it has considerably less available spaces since the roof and ground floor are not available for development in the present state, also because it has limited spaces and deteriorated facilities, and finally because the area’s socioeconomic status is on the lower side compared to the other buildings and in general. Lastly, the Ouzonnian building is in a place between the two buildings based on the evaluation, but it is closer in overall value to Little Venice due to its deteriorated facilities and infrastructure, as well as its poor architectural features, and finally due to its high environmental impact considering the building’s location being along a noisy street with high traffic and overall pollution. Ouzonnian still has a higher grade since it has much more available spaces with large areas, which provide higher flexibility to incorporate diverse functions or activities, and it also has open spaces and bigger openings, unlike Little Venice ( Selim, 2023). These factors gave it a higher overall grade regardless of whether Little Venice is a rich building considering its history, architectural features, and overall aesthetic, which make it one of the most iconic buildings in the city of Alexandria. The Little Venice building is in a prime location along the waterfront, but the potential for development is limited due to the lower number of available spaces, while the Ouzonnian building is in an arguably compromised location due to noise and pollution, but it has high traffic and more available spaces considering the BUA and the surrounding amenities, services, and high tourist presence in downtown Cairo compared to Alexandria.

**Figure 26.**
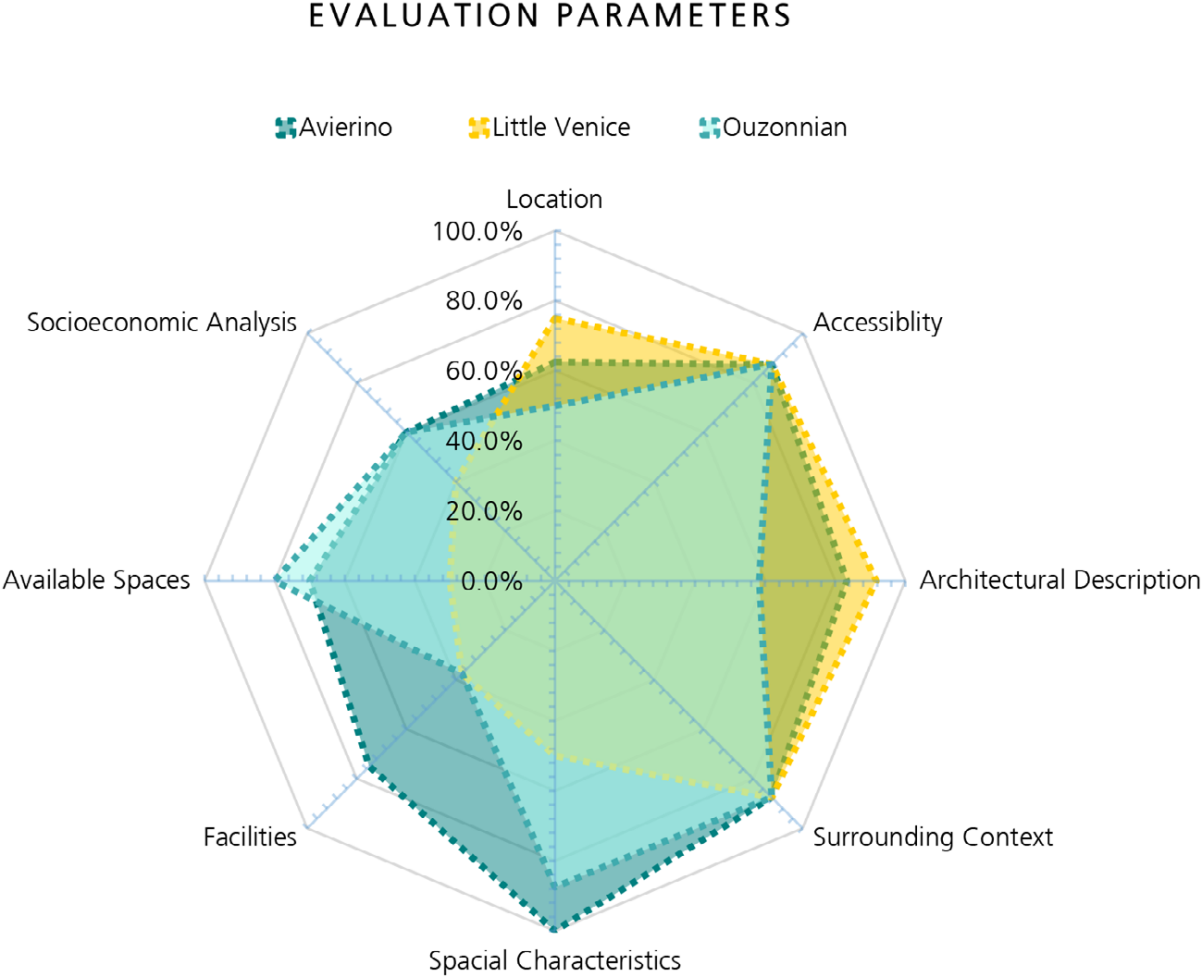
Radial diagram illustrates the evaluation of the three buildings and compares them to each other as a valuable comparison tool. Source: Author, 2023.

The radial diagram in Figure 26 shown above demonstrates the evaluation of the three buildings stacked on top of each other, showing the strengths and weaknesses of each parameter and how it compares to the remaining buildings. This diagram represents a tool that has a high potential for being utilized by investors and producers aiming to identify the most efficient investment, as it shows which building is most suitable considering the requirements of the developers or target users. This diagram helps investors formulate a brief idea about each building and decide which one would be worth the investment or which ones have a higher priority for acquisition. Finally, this diagram shows the common area where the three buildings all intersect, which defines the minimum or average values a heritage building must have in order to be considered for AR projects. For example, all of the buildings have a medium to high value considering the location, accessibility, architectural description, and surrounding context, while other values vary considerably between the buildings.

### Application on case studies

As the final step for this evaluative approach, the researcher applies the developed method to the designated case studies in order to apply the four-step decision-making framework as shown in Table 7 to identify the potential functions that could be introduced in each building, as well as the scale, type, and category of each function.

**Table 7.** Table demonstrates the application of the four decision-making phases and the decision based on the overall evaluation percentage on the three analyzed buildings. Source: Author, 2023.

Function								
Parameter	Location	Accessibility	Surrounding context	Spatial characteristics	Facilities	Available spaces	**Total**	**%**
AVI	5	7	7	8	6	7	**40/50**	**80%**
LV	6	7	7	4	3	3	**30/50**	**60%**
OUZ	4	7	7	7	3	8	**36/50**	**72%**
Recommended functions	**AVI**	Maximum potential for MUD, diverse high end functions could be incorporated						
	**LV**	Medium to high potential for MUD, but more hospitality services						
	**OUZ**	Maximum potential for MUD, diverse high end functions could be incorporated						
Scale								
Parameter	Spatial characteristics		Facilities		Available spaces		**Total**	**%**
AVI	8		6		7		**21/26**	**80.7%**
LV	4		3		3		**10/26**	**38.5%**
OUZ	7		3		8		**18/26**	**69.2%**
Recommended scale	**AVI**	Large scale projects could be introduced to the building						
	**LV**	Small scale projects due to existing limitations						
	**OUZ**	Medium to high scale projects						
Type								
Parameter	Location	Accessibility	Architectural description	Available spaces	Socioeconomic analysis		**Total**	**%**
AVI	5	7	10	7	6		**35/48**	**73%**
LV	6	7	11	3	4		**31/48**	**64.5%**
OUZ	4	7	7	8	6		**32/48**	**66.7%**
Recommended type	**AVI**	Premium hospitality, arts exhibitions, high-end retail, F&B, and offices						
	**LV**	Boutique hotels due to limited scale, mid-range retail, F&B, and offices						
	**OUZ**	B&B due to bigger scale, cultural spaces, mid-range retail, F&B, and offices						
Category								
Parameter	Location	Architectural description	Surrounding context		Socioeconomic analysis		**Total**	**%**
AVI	5	10	7		6		**28/38**	**73%**
LV	6	11	7		4		**28/38**	**73%**
OUZ	4	7	7		6		**24/38**	**63%**
Recommended category	**AVI**	High category of finishes, premium furnishing and equipment						
	**LV**	High category of finishes, premium furnishing and equipment						
	**OUZ**	Average category of finishes, standard furnishing and equipment						

Table 7 demonstrates the outcome of the final step of the evaluation process as an example that could potentially be applied to different buildings and cases in any adaptive reuse project, whether in Egypt or anywhere else. Depending on the building and its context, and the previously described parameters, those parameters form the main framework that any developer or stakeholder could utilize as a guideline for evaluating different buildings and deciding on whether the investment is feasible, in deciding what to invest in considering the building in general and the acquisitions within the building itself, also decide on the possible functions that could be introduced and the scale, type, and category of each of those functions. This evaluation also provides a detailed analysis of any building, as this analysis was the basis on which the researcher performed the final decision-making steps and provides a brief assessment of a building that could be presented to potential investors and other producers.

Throughout this research, the author determines that not all developments are required to incorporate all of the aforementioned components to form a balanced mixed-use development, but some minor functions can always be special and designated to certain destinations and hubs in order to form a healthy fabric on the urban scale and to give each property a certain edge over other properties and developments and also to maintain this cohesive network formed between buildings and each other in an area where functions complete and complement each other, the users could feel that the experience is timeless, doesn’t grow old or obsolete after a short time, and is self-sufficient, and developers always see adaptive reuse as a sustainable investment.

Furthermore, the researcher concludes that in the adaptive reuse industry, the role of different stakeholders such as the designer’s, has grown and evolved into a more holistic, multidisciplinary role that requires major knowledge in various fields; understanding the financial aspects of every project, such as the required budget and feasibility, is of critical importance to ensure a higher success rate for the project ( Drivers, 2013). Also, having a basic understanding of the legal aspects of development and acquisition of assets and spaces is important, as it has an impact on decision-making and the selection of functions, which then has an impact on the design approach ( Bottero *et al.,* 2019). Finally, property management teachings are fundamental in order to create successful programs, considering each function that was decided to be part of the component mix. All of the aforementioned disciplines have direct implications and influence most, if not all, of the design decisions in the adaptive reuse of heritage buildings and mixed-use developments.

## Conclusions

The researcher provides those findings as an extension to previous research and literature produced by other studies and aims that this study could assist other researchers and stakeholders involved in the industry with developing a brief understanding and knowledge based on practical experience in the adaptive reuse field, especially in developing countries. This research could potentially be a stepping-stone for the creation of a more developed and advanced method of evaluating heritage buildings and an innovative tool for decision-making parameters that could be resourcefully utilized by regulators and other stakeholders, ultimately for the goal of saving the remaining heritage that has been deteriorated, and in many cases lost, due to the negligence and ignorance of its value to the community and the cities they live in.

## Data Availability

The data from this study was provided from Sigma properties and all information about the buildings presented in the case studies including historical and present day photos of the buildings’ exterior and interior, floor plans, and drawings are included in the underlying and extended data alongside the results of this study with permission from Sigma. However, financial data underlying this study including financial figures and budgets is restricted as it is considered confidential information by the supplying entity. The author explains the restrictions on the data obtained from a third party as follows:
• Detailed historical data for buildings, such as drawings and ownership documents • Detailed project packages, such as tendering and design packages • Financial data and statistics for completed projects or budgets for upcoming projects. In order to access any of the aforementioned restricted data from the third party (Sigma Properties), a company representative shall be contacted via email at m.fathy@sigmaproperties.net with the required information along with the institution or organization details, and the reason for the request for access and information shall be provided accordingly. Alternatively, the author shall be contacted via correspondence email at ar.mohanned.selim@gmail.com for assistance with the supply of any information and coordination. The company withholds the right to refuse to disclose detailed financial information if deemed to cause a conflict of interest. In order to access the data obtained from the third party (Sigma Properties), the company’s website, http://sigmaproperties.net/, or the website, https://www.coterie-eg.com/, which represents the company’s development arm, should provide any publicly available data and general information regarding the properties, such as photos, historical background, architect information, and building features and spaces. Any reuse of the figures and photos used in this study must receive permission from Sigma Properties. Another means to access necessary information and intermediary data is by contacting the aforementioned company representative through email at m.fathy@sigmaproperties.net or through the author *via* the correspondence address ar.mohanned.selim@gmail.com. Mendeley data: The revitalization of endangered heritage buildings in developing countries: A Decision-making framework for investment and determining highest and best use in Egypt. https://doi.org/10.17632/998c7bf8jh.1. ( Selim, 2023). This dataset contains the following underlying data:
• Avierino building (case study 1). (Data for case study 1 used in this study including layout, developments, figures, and photos). • Little Venice building (case study 2). (Data for case study 2 used in this study including layout, developments, figures, and photos). • Ouzonnian building (case study 3). (Data for case study 3 used in this study including layout, developments, figures, and photos). • Axonometric diagrams for 3 case studies (source). (Axonometric diagrams for the 3 case studies and their sources). Data are available under the terms of the Creative Commons Attribution 4.0 International license (CC-BY 4.0). Mendeley data: The revitalization of endangered heritage buildings in developing countries: A Decision-making framework for investment and determining highest and best use in Egypt. https://doi.org/10.17632/998c7bf8jh.1. ( Selim, 2023). This dataset contains the following extended data:
• MAPS (photoshop source). (Maps of all locations in this study including source data). • Research figures & tables. (All figures and tables included in this manuscript). Data are available under the terms of the Creative Commons Attribution 4.0 International license (CC-BY 4.0).
