# Supplementary material for: The revitalization of endangered heritage buildings: A decision-making framework for investment and determining the highest and best use in Egypt
Source: F1000Res. 2023 Dec 19;12:874. Originally published 2023 Jul 24. [Version 2] doi: 10.12688/f1000research.135214.2 (PMC10904985; doi:10.12688/f1000research.135214.2)
Supplement: Peer review history

# Reviewer report: Reviewer response for version 2

**DOI:** 10.5256/f1000research.158799.r231383

**Published:** 2024-02-29

**Authors:** Waled Shehata

**Affiliations:** 1 Faculty of Society and Design, Bond University, Robina, Queensland, Australia

The reviewer would like to thank the authors on the efforts they undertook to develop and enhance this piece of work.

 I confirm many of the remarks have been considered, references updated, arguments well reformulated..etc. However, I recommend the below few tweaks.

**Comments in line with the first revision:**

 1 - The *Tendering and Contractor Selection* phase is maybe not so relevant as agreed by the authors and the reviewer. I strongly recommend eliminating this section.

 2 - Space Syntax was only a recommendation. No need to apply it in this research, but implications for using it in future research can be useful.

 3 - Confirming previous comments, the assumption that *“projects were utilised by the researcher as a reference due to multiple factors such as functional and economic viability, preservation of heritage value, community engagement and stakeholder collaboration, and finally, the recognition of the local community”* still needs backing up with references, even just as quick and short in-text citation references. This claim is a cornerstone of the validity of this paper and deserves to be supported.

Is the case presented with sufficient detail to be useful for teaching or other practitioners?

Partly

Is the work clearly and accurately presented and does it cite the current literature?

No

If applicable, is the statistical analysis and its interpretation appropriate?

Not applicable

Are all the source data underlying the results available to ensure full reproducibility?

Yes

Are the conclusions drawn adequately supported by the results?

No

Is the background of the case’s history and progression described in sufficient detail?

No

Reviewer Expertise:

Heritage Adaptive Reuse-Uncomfortable heritage-Historic Cairo-Heritage Conservation and Management in NSW, Australia

I confirm that I have read this submission and believe that I have an appropriate level of expertise to confirm that it is of an acceptable scientific standard.

# Reviewer report: Reviewer response for version 1

**DOI:** 10.5256/f1000research.148318.r202843

**Published:** 2023-09-19

**Authors:** Tianchen Dai

**Affiliations:** 1 School of Design, East China Normal University, Shanghai, Shanghai, China

This is a very interesting article that provides a framework for developing legacy assets and investment criteria to help investors, designers and stakeholders prepare projects and measure their performance. It is always difficult to combine architectural practice with research, and this study demonstrates a nice attempt.

 I have a couple of minor suggestions to help improve the quality of this paper before it is ready for indexing:

1. The introduction is rather brief. The authors should extend this section substantially with a thorough literature review and background on this study. The aims and objectives of the study can be incorporated into the Introduction.
2. Some very long sentences should be cut into shorter ones or rewritten. For example, the last sentence in Section 'Research aims and objectives.
3. In Table 1, the developed parameters and sub-parameters are listed by the authors. It would be more convincing to explain more about how these parameters were developed, based on rigorous literature reviews or preliminary studies.
4. Considering Table 3, where is the data to support the results in this table? The authors should clearly show the analysis procedure, if not in the main section, at least in the appendix.

Is the case presented with sufficient detail to be useful for teaching or other practitioners?

Yes

Is the work clearly and accurately presented and does it cite the current literature?

Partly

If applicable, is the statistical analysis and its interpretation appropriate?

Partly

Are all the source data underlying the results available to ensure full reproducibility?

Yes

Are the conclusions drawn adequately supported by the results?

Yes

Is the background of the case’s history and progression described in sufficient detail?

No

Reviewer Expertise:

Architecture; urban studies; cultural heritage; city branding

I confirm that I have read this submission and believe that I have an appropriate level of expertise to confirm that it is of an acceptable scientific standard.

# Reviewer report: Reviewer response for version 1

**DOI:** 10.5256/f1000research.148318.r198498

**Published:** 2023-09-04

**Authors:** Waled Shehata

**Affiliations:** 1 Faculty of Society and Design, Bond University, Robina, Queensland, Australia

The reviewer would like to thank the authors of this paper. The selected topic is much needed in Egypt where, indeed, heritage gets delisted and demolished, or undergo destructive conservation works under the miserable State-branded ‘development’.

 The research objective which is to develop a framework for heritage adaptive reuse project to increase chances of achieving economically viable developments and best use is a noble objective. However, the intent is lost due to many strategic issues as listed below.

**Strategic issues:**

 The targeted heritage buildings’ category(ies) for this study is not specified. For instance, does it target those listed as monument of Ministry of Tourism and Antiquities, National Organisation for Urban Harmony (NOUH), and/or other identified to have heritage significance by heritage specialists…etc. Different grade of listings of heritage buildings in Egypt have different acceptable levels of intervention for their adaptive reuse, conservation and management. The three selected case studies are also in different regions in Egypt and are of different construction periods and styles. Heritage buildings’ size, level of significance, age and construction system would most likely limit the effectiveness of this framework. It is of high importance for this framework to be sound is to specifically target a group of heritage buildings of homogeneous characteristics. Otherwise if the authors intend to exclusively target heritage buildings such as those already examined, their common characteristics needs to be clearly spelled out (i.e. multi-storey, concrete structural system, listed in NOUH…etc) while providing a short background on the statutory requirements/challenges for their reuse in the Egyptian heritage planning laws according to their listing(s). Once this is done, the research intent would be clearer and the framework would be better targeted and usable.

 The references are relatively old. With only a couple of references published in 2019–which is still not very recent, most references range between 2005-2016. Referring to recent literature is imperative and I am certain would greatly assist refine the article and the proposed framework.

 The research aim is not well formulated. Using references in the objective is not justified. The objective should be in your own words unless these references recommend such objective, something that need to be spelled out to be clear. Additionally, please use shorter sentences to be understandable. Once this is done, the objective would sound more focused and achievable.

 Some terms suddenly pop-up in the article without proper explanation or if they were used in literature. Terms and assessable factors which suddenly appear in the article include ‘highest and best use’, ‘function, type, category and scale’, ‘success of mixed-use developments’ and others. The research readily perceive some of these factors as a virtue/advantage but without supporting this argument with one sentence or a credible reference.

 In P4, the authors wrote: ‘completed adaptive reuse (AR) scenarios’. ‘Completed’ here is very vague. Do the authors mean the work is completed, or the buildings are operational, …etc. Also this instance is the first use of the word ‘scenarios’, which confuses the readers. You may need to carefully choose the terms you use in the article to be connected to the flow of ideas and consistent throughout the article.  

 The second paragraph in page 4 explains a usual process of architectural project planning and redevelopment with only a few minor mention to heritage AR peculiarities here and there. I believe it would be more space saver if the authors bluntly say that heritage AR projects are similar to any other architecture refurbishment project with the differences of x,y and z. Multiple refurbishment project processes are also noted in page 11 being Tender and contractor selection and Operation and evaluation, which are irrelevant to the article’s objective and can be eliminated without the slightest effect the quality of the framework.

 The amount of surveyed literature does not match the intended purpose. To generate a working and valid framework, one usually need to examine a considerable number of literature. Such survey would lead to deducing and\or inducing a number of factors that are relevant to the research objective. These are referred to here as ‘parameters’. To help the authors in their literature review, they may consider these questions: Are these parameters dependant or independent variables? What primary sources support them being important enough to be considered in this research? What do the authors mean by ‘different’ contemporary literature? How *different* these literature are classified?…etc.

 I strongly recommend undertaking a targeted literature review (LR) on the success factors of similar projects of heritage adaptive reuse in Egypt while revising this research. The volume of research already existing on this topic in Egypt is enormous and I am certain it will better inform this research. I am aware most of them are not free on the web, but please consider reaching out for assistance if the AASTMT does not provide access to researchers. A LR in the first section of this article would better inform gaps in adaptive reuse process which would enable your research outcome to be more sound.

 The reason three case studies were selected is not mentioned except in page 33 where the article is closing up. The intent need to be mentioned much earlier, nonetheless, it needs to be well articulated. If these case studies are used to evaluate the framework, you need to explain if these case studies are representative of the heritage buildings in Egypt, why were they are used to test the framework, and other reasons for their selection. Each reason may then require different handling of the case studies and the way their data is analysed and presented. Clearly defining the reason these case studies were selected and formulating the discussion accordingly is crucial to achieve the validity and applicability of the proposed framework.

 Also related to the previous point, the research mentioned: “projects were utilised by the researcher as a reference due to multiple factors such as functional and economic viability, preservation of heritage value, community engagement and stakeholder collaboration, and finally, the recognition of the local community.” How did they achieve this to be selected at the first place? What literature including media and news shared this point of view? More clarification is required.

 Additionally, I would argue that selection of three case studies by the same developer (Sigma properties) highly reduces confidence in the generalisability of the proposed framework. Selecting all projects that are realised by the same developer fails to put the framework in test in adaptive reuse projects done by other types of project-driving stakeholders such as government entities-which, by virtue, should target economic viability, international and national cultural institutions—such as Bibliotheca Alexandria… and others.

 The generalisability of the research is highly questionable. Cairo and Alexandria neither represent Egypt nor the case studies represent developing countries. More careful consideration while referring to the locality of study would be useful, such as simply focusing on Cairo, Alexandria or both, and removing ‘developing countries’ and ‘Egypt’ from the article including the title. Of course, every choice would require changes in the body of the article to best respond to the research focus.

 In the conclusion, the authors used a bit harsh statements such as “ignorance … to the community”. Kindly revise.

 Long sentences in the conclusion are unclear and ambiguous. Kindly rewrite.

 In page 4, ‘unique features’ is not a common term in heritage conservation field. Perhaps you need to refer to Assessing heritage significance: Guidelines for assessing places and objects against the Heritage Council of NSW criteria to familiarise yourself with the terms used in the context of heritage AR. Reference link: https://www.environment.nsw.gov.au/-/media/OEH/Corporate-Site/Documents/Heritage/assessing-heritage-significance-guidelines-assessing-places-objects-against-criteria-230167.pdf

 In page 29, rewrite the 4 ^th^ paragraph to be understandable and clearer.

 Last but not least, the academic English used is not consistent and some of the terms are not clear, i.e. Target market and the use of past tenses in many occurrences. Nonetheless, long sentences lose their objective and turn to vague statements. The writing style is literal translation from Arabic, something that requires experienced rewriting of many sections of the article.

**Minor issues:**

 I was hoping that the authors would consider clarifying what would be the impact of an architectural competition for the design of the AR would make on the framework, if any.

 The article is considerably lengthy. Consider removing unnecessary sections such as: Students and educators versions of programs, free alternative software suggestions, list of abbreviations (you can just use abbreviation following first mention of the full term), Tendering and Contractor Selection Operation and Evaluation as well as other conventional project phases.

 In Table 1, consider adding Public transport to the Diverse modes of transport.

 Also consider combining Architectural description and Spatial characteristics.

 Consider using Space Syntax for analysing the spatial layouts and functional programs for the AR of the heritage buildings. Space Syntax may contribute to the quality of the functional usability of the buildings.

 In the Maps prepared for the case studies, it is imperative to provide a scale. Alternatively, you may use walkability distances via annotated rings/circles. This is important to assist readers understand the sites’ proximity to elements of the urban surroundings. You may also consider distinguishing the location of the case study building with a different colour and providing a map key.

 In Table 2, kindly elaborate on the impact of whether the building being heritage listed.

 In page 4, how does Merlino 2018 explain your own preferences of the selected case studies. Kindly revise this section.

 In the first sentence in the conclusion, kindly explain ‘those’ findings.

Is the case presented with sufficient detail to be useful for teaching or other practitioners?

Partly

Is the work clearly and accurately presented and does it cite the current literature?

No

If applicable, is the statistical analysis and its interpretation appropriate?

Not applicable

Are all the source data underlying the results available to ensure full reproducibility?

Yes

Are the conclusions drawn adequately supported by the results?

No

Is the background of the case’s history and progression described in sufficient detail?

No

Reviewer Expertise:

Heritage Adaptive Reuse-Uncomfortable heritage-Historic Cairo-Heritage Conservation and Management in NSW, Australia

I confirm that I have read this submission and believe that I have an appropriate level of expertise to state that I do not consider it to be of an acceptable scientific standard, for reasons outlined above.

## References

[1] : Towards the comprehensive and systematic assessment of the adaptive reuse of Islamic architectural heritage in Cairo. *Journal of Cultural Heritage Management and Sustainable Development* .2015;5(1) : 10.1108/JCHMSD-02-2014-0003 14-29 10.1108/JCHMSD-02-2014-0003

# Response

**Published:** 2023-11-10

**Authors:** Mohanned Selim

**The authors would like to thank you for your valuable input and careful inspection of this research, your effort is much appreciated.**

**Strategic issues:**

*The targeted heritage buildings’ category(ies) for this study is not specified. For instance, does it target those listed as monument of Ministry of Tourism and Antiquities, National Organisation for Urban Harmony (NOUH), and/or other identified to have heritage significance by heritage specialists…etc. Different grade of listings of heritage buildings in Egypt have different acceptable levels of intervention for their adaptive reuse, conservation and management. The three selected case studies are also in different regions in Egypt and are of different construction periods and styles. Heritage buildings’ size, level of significance, age and construction system would most likely limit the effectiveness of this framework. It is of high importance for this framework to be sound is to specifically target a group of heritage buildings of homogeneous characteristics. Otherwise if the authors intend to exclusively target heritage buildings such as those already examined, their common characteristics needs to be clearly spelled out (i.e. multi-storey, concrete structural system, listed in NOUH…etc) while providing a short background on the statutory requirements/challenges for their reuse in the Egyptian heritage planning laws according to their listing(s). Once this is done, the research intent would be clearer and the framework would be better targeted and usable.*

 Regarding the listing of the buildings, the indication of this has been added to the introduction section of the comparative analysis and a justification of this has been explained as follows: the first two cases of Avierino and Little Venice are listed as heritage type B which means that those assets are protected by law and indicates that interior modifications are permissible while maintaining the exterior facades, as for Ouzonnian building, it is not listed as heritage but this is justified by its historical/cultural significance which is evident through its architecture, architect, and context. This building was once argued to be the tallest building in downtown Cairo due its modern construction techniques which were considerably new during the time of construction, also Sayed Karim, the architect, was one of the leading figures in the field of Architecture in Egypt, the building has many stories and plays part in the narrative that represents the spirit of downtown Cairo. The justification in this is that the importance and significance of such buildings does not always have to be justified by their listing as heritage which has been evident through the years were heritage buildings with outstanding importance and significance were deteriorated and demolished due to their removal from the heritage listing or due to their nonexistence in the first place.

 Finally, regarding the justification for selecting the aforementioned buildings, this was based on the fact that the author has worked on the development of those buildings personally alongside a team of experts in the field, which perhaps needed to be clarified in the paper, also the selection of buildings with variable architectural styles, design, components, context, and location, was argued by the authors to add more validation to the framework being applicable to different buildings and producing successful results which were evident in those 3 cases considering revenues (indicated in the last section of each case), asset value, historical and cultural significance.

*The references are relatively old. With only a couple of references published in 2019–which is still not very recent, most references range between 2005-2016. Referring to recent literature is imperative and I am certain would greatly assist refine the article and the proposed framework.*

 The authors agree that this is of great importance to this study and more recent literature has been referenced in the article, many of which were related to framework development as advised. The references are as follows:

 Albu, S. 2021. The Economic Value and Valuation of Architectural Heritage. Journal of Building Construction and Planning Research, 9, 1-11. DOI: 10.4236/jbcpr.2021.91001. Available from: https://doi.org/10.4236/jbcpr.2021.91001.

 Armstrong, G., Wilkinson, S., & Cilliers, E. 2023. A framework for sustainable adaptive reuse: understanding vacancy and underuse in existing urban buildings. Frontiers in Sustainable Cities, 5. DOI: 10.3389/frsc.2023.985656.

 Fitri, I., Siregar, F., Silvana, S., Ariffin, N., Ginting, R., & Indira, S. 2023. An Adaptive Reuse Development Through Highest and Best Use Assessment: Case Study the Ex-Warenhuis, Medan, Indonesia. IOP Conference Series: Earth and Environmental Science, 1188, 012045. DOI: 10.1088/1755-1315/1188/1/012045.

 Hamida, M. 2020. Development of a Framework for the Effective Implementation of Building Adaptive Reuse throughout the Project Life-Cycle in Saudi Arabia. DOI: 10.13140/RG.2.2.15488.35840.

 Haroun, H., Bakr, A., & Hasan, A. 2019. Multi-criteria decision making for adaptive reuse of heritage buildings: Aziza Fahmy Palace. Alexandria University Alexandria Engineering Journal.

 NSW Environment. 2022. Investigating Heritage Significance [online]. NSW Environment. Available from: https://www.environment.nsw.gov.au/research-and-publications/publications-search/investigating-heritage-significance [Accessed 06 Oct 2023].

 NSW Government. 2021. Investigating Heritage Significance: A guide to identifying and examining heritage items in NSW. NSW Government through the Heritage Council of NSW.

 Pasha, C., Dewi, C., & Djamaludin, M. 2021. Adaptive reuse of old houses as coffee shop: Environmental and spatial aspects. IOP Conference Series: Earth and Environmental Science, 881, 012046. DOI: 10.1088/1755-1315/881/1/012046.

 Vehbi, B.O., Günçe, K., & Iranmanesh, A. 2021. Multi-Criteria Assessment for Defining Compatible New Use: Old Administrative Hospital, Kyrenia, Cyprus. Sustainability, 13, 1922. DOI: 10.3390/su13041922.

*The research aim is not well formulated. Using references in the objective is not justified. The objective should be in your own words unless these references recommend such objective, something that need to be spelled out to be clear. Additionally, please use shorter sentences to be understandable. Once this is done, the objective would sound more focused and achievable.*

 References were removed from the objective as advised and the sentences were made shorter for legibility.

*Some terms suddenly pop-up in the article without proper explanation or if they were used in literature. Terms and assessable factors which suddenly appear in the article include ‘highest and best use’, ‘function, type, category and scale’, ‘success of mixed-use developments’ and others. The research readily perceive some of these factors as a virtue/advantage but without supporting this argument with one sentence or a credible reference.*

 Explanation for those terms has been added to the Methods section when first mentioned as advised, and their virtue/advantage has been supported as well.

*In P4, the authors wrote: ‘completed adaptive reuse (AR) scenarios’. ‘Completed’ here is very vague. Do the authors mean the work is completed, or the buildings are operational, …etc. Also this instance is the first use of the word ‘scenarios’, which confuses the readers. You may need to carefully choose the terms you use in the article to be connected to the flow of ideas and consistent throughout the article.*

 Yes, the authors mean that the proposed adaptive reuse models developed through the framework are evidently successful based on the fact that they have applied the AR model and provided significant revenues and returns, on Investment and on Asset, and they repositioned the buildings’ significance to the community. This has been explained in the article as advised and the word scenarios was changed to models to avoid confusion.

*The second paragraph in page 4 explains a usual process of architectural project planning and redevelopment with only a few minor mention to heritage AR peculiarities here and there. I believe it would be more space saver if the authors bluntly say that heritage AR projects are similar to any other architecture refurbishment project with the differences of x,y and z. Multiple refurbishment project processes are also noted in page 11 being Tender and contractor selection and Operation and evaluation, which are irrelevant to the article’s objective and can be eliminated without the slightest effect the quality of the framework.*

 The aforementioned processes were explained in the article as phases of the Adaptive Reuse project from start to finish, or delivering the project and evaluating its operation, and while the tendering and contractor selection phase might indeed be irrelevant but the operation phase was justified by the authors as this is when the projects’ performance undergoes evaluation and assessment which provides feedback and proof of the framework’s success, or failure. Please advise if this is not valid.

*The amount of surveyed literature does not match the intended purpose. To generate a working and valid framework, one usually need to examine a considerable number of literature. Such survey would lead to deducing and\or inducing a number of factors that are relevant to the research objective. These are referred to here as ‘parameters’. To help the authors in their literature review, they may consider these questions: Are these parameters dependant or independent variables? What primary sources support them being important enough to be considered in this research? What do the authors mean by ‘different’ contemporary literature? How different these literature are classified?…etc.*

 These parameters are evaluated independently based on the sub-parameters as seen in Table 1, however they are dependent since they are treated as a collective of variables forming an overall general evaluation grade as seen in Table 6.

 The term ‘different’ indicates different types of sources (Primary, secondary, and tertiary sources) but this term was removed due to being unnecessary.

*I strongly recommend undertaking a targeted literature review (LR) on the success factors of similar projects of heritage adaptive reuse in Egypt while revising this research. The volume of research already existing on this topic in Egypt is enormous and I am certain it will better inform this research. I am aware most of them are not free on the web, but please consider reaching out for assistance if the AASTMT does not provide access to researchers. A LR in the first section of this article would better inform gaps in adaptive reuse process which would enable your research outcome to be more sound.*

 Your assistance in this matter, if convenient, would be much appreciated as several literature was indeed reviewed however many of the examined open-source data was irrelevant, if it would be possible to recommend some of the sources you mentioned or help with access it would be of immense support.

*The reason three case studies were selected is not mentioned except in page 33 where the article is closing up. The intent need to be mentioned much earlier, nonetheless, it needs to be well articulated. If these case studies are used to evaluate the framework, you need to explain if these case studies are representative of the heritage buildings in Egypt, why were they are used to test the framework, and other reasons for their selection. Each reason may then require different handling of the case studies and the way their data is analysed and presented. Clearly defining the reason these case studies were selected and formulating the discussion accordingly is crucial to achieve the validity and applicability of the proposed framework.*

 The authors agree that this is necessary for the validity of the proposed framework, and this has been applied and explained in the first question in this document. The authors tried their best to achieve this as advised, nevertheless, the argument of making this study/framework as simplified as possible, which was one of the main goals of this article, to facilitate and simplify the redevelopment of heritage assets in Egypt and present a legible guide for different stakeholders who have the potential of making change. Please advise if you require any further clarification or changes.

*Also related to the previous point, the research mentioned: “projects were utilised by the researcher as a reference due to multiple factors such as functional and economic viability, preservation of heritage value, community engagement and stakeholder collaboration, and finally, the recognition of the local community.” How did they achieve this to be selected at the first place? What literature including media and news shared this point of view? More clarification is required.*

 As for functional and economic viability, the successful operation and revenues provided by those projects was one of the main reasons for selection, however, the financial data was not permissible for disclosure in this research and are confidential to the developer but were shared with the author.

 Preservation of heritage value was achieved through the restoration and preservation of original designs and features, and through how those projects highlighted the history of the building, its tenants, and the architect.

 Community engagement and Recognition of the community was achieved through the traffic visiting those buildings and engagement in various events, exhibitions, and tours. Also, research and documentation performed by various researchers and experts in the field.

 Stakeholder collaboration was achieved through working along experts such as consultants, operators, investors, and designers in the preparation, execution, and operation phases of those projects. This wasn’t clarified in detail throughout the article to avoid making it longer than it is but was indicated by the authors for possible inquiry by interested persons or researchers through contacting the corresponding author or the owner / developer (Sigma Properties, Coterie).

*Additionally, I would argue that selection of three case studies by the same developer (Sigma properties) highly reduces confidence in the generalisability of the proposed framework. Selecting all projects that are realised by the same developer fails to put the framework in test in adaptive reuse projects done by other types of project-driving stakeholders such as government entities-which, by virtue, should target economic viability, international and national cultural institutions—such as Bibliotheca Alexandria… and others.*

 The reason behind this is that the author was part of the team that worked on the redevelopment of those 3 buildings, the authors believe that this hands-on experience and professional involvement actually adds more credibility and an accurate view and assessment of the performance of those AR projects. I completely agree that this creates some kind of conflict and that was a point of discussion when preparing this article, but the authors came to an agreement that the aforementioned role of the author adds to the validity and practicality in which this study was based on.

*The generalisability of the research is highly questionable. Cairo and Alexandria neither represent Egypt nor the case studies represent developing countries. More careful consideration while referring to the locality of study would be useful, such as simply focusing on Cairo, Alexandria or both, and removing ‘developing countries’ and ‘Egypt’ from the article including the title. Of course, every choice would require changes in the body of the article to best respond to the research focus.*

 The authors agree that this is indeed questionable; the author added to the conclusion as part of the recommendations that the proposed framework has the potential of application in developing countries or other contexts where AR is required or studied, however this has not been tested or proven successful in different contexts so this would need to be tested first, and future research could modify or add to the proposed framework in order to suit said different contexts.

 Regarding the title, I’m not sure if I have the ability to change as per F1000 guidelines but I will attempt to make this change.

*In the conclusion, the authors used a bit harsh statements such as “ignorance … to the community”. Kindly revise.*

 This has been revised and the term was changed to “unawareness”.

*Long sentences in the conclusion are unclear and ambiguous. Kindly rewrite.*

 This has been rewritten as advised.

*In page 4, ‘unique features’ is not a common term in heritage conservation field. Perhaps you need to refer to Assessing heritage significance: Guidelines for assessing places and objects against the Heritage Council of NSW criteria to familiarise yourself with the terms used in the context of heritage AR. Reference link: https://www.environment.nsw.gov.au/-/media/OEH/Corporate-Site/Documents/Heritage/assessing-heritage-significance-guidelines-assessing-places-objects-against-criteria-230167.pdf*

 This has been revised and the term was changed to ‘fabric’ as advised based on the provided reference.

*In page 29, rewrite the 4 ^th^ paragraph to be understandable and clearer.*

 This paragraph has been rewritten as advised.

*Last but not least, the academic English used is not consistent and some of the terms are not clear, i.e. Target market and the use of past tenses in many occurrences. Nonetheless, long sentences lose their objective and turn to vague statements. The writing style is literal translation from Arabic, something that requires experienced rewriting of many sections of the article.*

 The aforementioned unclear terms have been clarified or modified wherever possible but please advise if this still needs revision. Please note that the author’s background must definitely have some kind of influence on the writing style, knowing that the use of translating services (such as Google Translate) of paragraphs or parts of this study was never done, translations of some Arabic terms or words has, but never to full sentences or paragraphs. Please let us know if there are any other critical issues to any specific paragraph which requires rewriting, aside from the ones you requested in the reviewer report as those were rewritten, unless they require further modifications.

**Minor issues:**

*I was hoping that the authors would consider clarifying what would be the impact of an architectural competition for the design of the AR would make on the framework, if any.*

 This will be analyzed in depth in future studies. However, a competition as such would definitely have an impact on the developed framework as it would provide valuable feedback concerning design aspects and it would provide valuable insight on the developed parameters and potential modifications or additions when assessing the produced projects.

*The article is considerably lengthy. Consider removing unnecessary sections such as: Students and educators versions of programs, free alternative software suggestions, list of abbreviations (you can just use abbreviation following first mention of the full term), Tendering and Contractor Selection Operation and Evaluation as well as other conventional project phases.*

 The sections you mentioned are required by F1000 so I believe it won’t be possible to remove them as the editors specifically requested their addition. As for project phases, this was explained previously but please advise if invalid.

*In Table 1, consider adding Public transport to the Diverse modes of transport.*

 Public transport is indeed the most important factor in this sub-parameter, which was indicated through the case studies analysis, but this will be indicated in the table as advised.

*Also consider combining Architectural description and Spatial characteristics.*

 Both of those parameters were differentiated by the authors as follows: The Architectural Description represents all that is related to the architectural aspects of the building such as the style influenced by the design features and ornaments, the listing of the building and the condition of said features, the architect and the story of the property or narrative derived from the history of the surrounding context in relation to the aforementioned elements.

 On the other hand, the Spatial Characteristics parameter indicates items related to the structural system and its condition, the availability of spaces within the building, whether this building is fully empty or if it has specific floors or units that are available, and this availability differs as demonstrated in the axonometric diagrams in the case studies analysis, and finally the spatial limitations such as the heights, sizes of openings, and court areas.

*Consider using Space Syntax for analysing the spatial layouts and functional programs for the AR of the heritage buildings. Space Syntax may contribute to the quality of the functional usability of the buildings.*

 This will be studied and any information with regards to the mentioned technique would be valuable.

*In the Maps prepared for the case studies, it is imperative to provide a scale. Alternatively, you may use walkability distances via annotated rings/circles. This is important to assist readers understand the sites’ proximity to elements of the urban surroundings. You may also consider distinguishing the location of the case study building with a different colour and providing a map key.*

 The building is currently indicated with a different color to easily distinguish it. Adding walkability distances was not feasible due to technical difficulties.

*In Table 2, kindly elaborate on the impact of whether the building being heritage listed.*

 This elaboration has been added to the architectural description parameter as advised.

*In page 4, how does Merlino 2018 explain your own preferences of the selected case studies. Kindly revise this section.*

 The intention here is referring to Merlino’s approach in selecting the case studies, Merlino selected the case studies in his study based on various factors and the authors followed a similar approach.

*In the first sentence in the conclusion, kindly explain ‘those’ findings.*

 ‘Those’ refers to the developed framework and the parameters forming the evaluation model.
